# Secretory Carrier Membrane Protein 3 Interacts with 3A Viral Protein of Enterovirus and Participates in Viral Replication

**DOI:** 10.1128/spectrum.00475-21

**Published:** 2021-08-11

**Authors:** Jia-Ying Lu, Gary Brewer, Mei-Ling Li, Kai-Zhe Lin, Chien-Chih Huang, Li-Chen Yen, Jing-Yi Lin

**Affiliations:** a Department of Clinical Laboratory Sciences and Medical Biotechnology, College of Medicine, National Taiwan University, Taipei, Taiwan; b Department of Biochemistry & Molecular Biology, Rutgers Robert Wood Johnson Medical School, Piscataway, New Jersey, USA; c Department of Medical Biotechnology and Laboratory Science, College of Medicine, Chang Gung University, Taoyuan, Taiwan; d Department of Microbiology and Immunology, National Defense Medical Center, Taipei, Taiwan; e Department of Laboratory Medicine, National Taiwan University Hospital, Taipei, Taiwan; Wright State University

**Keywords:** 3A protein, PI4KIIIβ, PI4P, SCAMP3, enterovirus, replication complex

## Abstract

Picornaviruses are a diverse and major cause of human disease, and their genomes replicate with intracellular membranes. The functionality of these replication organelles depends on the activities of both viral nonstructural proteins and co-opted host proteins. The mechanism by which viral-host interactions generate viral replication organelles and regulate viral RNA synthesis is unclear. To elucidate this mechanism, enterovirus A71 (EV-A71) was used here as a virus model to investigate how these replication organelles are formed and to identify the cellular components that are critical in this process. An immunoprecipitation assay was combined with liquid chromatography-tandem mass spectrometry (LC-MS/MS) analysis to identify 172 cellular proteins and four viral proteins associating with viral 3A protein. Secretory carrier membrane protein 3 (SCAMP3) was one of the host proteins we selected for further investigation. Here, we demonstrate by immunoprecipitation assay that SCAMP3 associates with 3A protein and colocalizes with 3A protein during virus infection. SCAMP3 knockdown or knockout in infected cells decreases synthesis of EV-A71 viral RNA, viral proteins, and viral growth. Furthermore, the viral 3A protein associates with SCAMP3 and phosphatidylinositol-4-kinase type III β (PI4KIIIβ) as shown by immunoprecipitation assay and colocalizes to the replication complex. Upon infection of cells with a SCAMP3 knockout construct, PI4KIIIβ and phosphatidylinositol-4-phosphate (PI4P) colocalization with EV-A71 3A protein decreases; viral RNA synthesis also decreases. SCAMP3 is also involved in the extracellular signal-regulated kinase (ERK) signaling pathway to regulate viral replication. The 3A and SCAMP3 interaction is also important for the replication of coxsackievirus B3 (CVB3). SCAMP3 also associates with 3A protein of CVB3 and enhances viral replication but does not regulate dengue virus 2 (DENV2) replication. Taken together, the results suggest that enterovirus 3A protein, SCAMP3, PI4KIIIβ, and PI4P form a replication complex and positively regulate enterovirus replication.

**IMPORTANCE** Virus-host interaction plays an important role in viral replication. 3A protein of enterovirus A71 (EV-A71) recruits other viral and host factors to form a replication complex, which is important for viral replication. In this investigation, we utilized immunoprecipitation combined with proteomics approaches to identify 3A-interacting factors. Our results demonstrate that secretory carrier membrane protein 3 (SCAMP3) is a novel host factor that associates with enterovirus 3A protein, phosphatidylinositol-4-kinase type III β (PI4KIIIβ), and phosphatidylinositol-4-phosphate (PI4P) to form a replication complex and positively regulates viral replication. SCAMP3 is also involved in the extracellular signal-regulated kinase (ERK) signaling pathway to regulate viral replication.

## INTRODUCTION

The *Enterovirus* genus is a member of the *Picornaviridae* family, which is a group of nonenveloped, positive-strand RNA viruses, including numerous important human pathogens. Well-known enteroviruses include poliovirus (PV), which causes paralytic poliomyelitis, and the human rhinoviruses, which cause the common cold and exacerbate asthma and chronic obstructive pulmonary disease (1, 2). Coxsackievirus B3 has also been implicated in chronic myocarditis and type I diabetes (3, 4). Another important member of the *Enterovirus* genus is enterovirus A71 (EV-A71), of which large outbreaks have occurred in the Asia-Pacific region in recent years (5). Infection by EV-A71 can result in hand-foot-and-mouth disease (HFMD) and herpangina. Children under 5 years old are particularly susceptible to the most severe forms of EV-A71-associated neurological complications, including aseptic meningitis, brainstem and/or cerebellar encephalitis, myocarditis, acute flaccid paralysis, and rapid fatal pulmonary edema and hemorrhage (6). Enterovirus D68 (EV-D68) is another globally reemerging pathogen. A recent outbreak in the United States is the largest one to be associated with severe respiratory illness and neurological complications (7). No antiviral drug for treating enterovirus infections has been approved. Vaccines against PV are available, but the WHO campaign for the eradication of poliomyelitis is encountering major problems as a result of the continual emergence of pathogenic, vaccine-derived revertants or recombinants. The development of vaccines against the other enteroviruses is practically impossible owing to the very large number of serotypes. Consequently, antiviral drugs are urgently required to combat enterovirus infections.

The enterovirus genome encodes four structural capsid proteins (VP1, VP2, VP3, and VP4) that facilitate cellular entry and delivery of the viral genome into the cytosol of the host cell; seven nonstructural proteins (2A^pro^, 2B, 2C, 3A, 3B, 3C^pro^, and 3D^pol^) mediate viral RNA replication (8). The enterovirus 3A protein has a length of 87 amino acids and exhibits membrane-targeting properties, because its C terminus contains a hydrophobic domain. The C-terminal domain is responsible for direct membrane association and has been demonstrated by molecular genetic studies to be important for viral replication (9–11).

All positive-strand RNA viruses induce the remodeling of cellular membranes to generate a scaffold for genomic RNA replication. These structures increase the local concentration of the viral and cellular cofactors that are required for replication and provide a protected environment that inhibits the recognition of virus proteins and nucleic acid by the innate immune system. Positive-strand RNA viruses can form replication organelles from various cytosolic membrane sources, such as the endoplasmic reticulum (ER), Golgi apparatus, endosomes and/or lysosomes, and mitochondria; the respective mechanisms of formation probably vary (12–14). Enteroviruses usually require cellular Golgi membranes to assemble replication organelles, which contain viral proteins, the viral RNA genome, and host factors, to amplify their genome. The development and functioning of these cellular membrane structures depend on rewiring cellular pathways into new configurations that are induced and regulated by viral proteins and host factors (13, 15). The enteroviral nonstructural proteins 2B, 2C, and 3A and the cleavage intermediates 2BC and 3AB are membrane-associated proteins and have been implicated in the formation of replication organelles (16, 17). In the early stages of poliovirus infection, viral protein 2B colocalizes with COP-II-coated vesicles, which bud from ER exit sites (18). The 3A proteins of PV and coxsackievirus B3 (CVB3) can perturb the cellular secretory pathway (19). One of the viral precursor proteins that is present during viral replication is 3AB, whose presumed function is that of the primer (3B) in viral RNA synthesis during infection (20, 21).

Reorganization of cellular membranes for enterovirus replication is thought to depend on cellular factors. Previous investigations have demonstrated that the 3A protein is responsible for the reorganization of membranes in the formation of viral replication organelles (16, 22). Viral 3A protein interacts with GBF1 (Golgi brefeldin A-resistant guanine nucleotide exchange factor 1), which is a GEF (guanine nucleotide exchange factor) for Arf1 (ADP-ribosylation factor 1), which is involved in recruiting COP-I coats to membranes in uninfected cells. The interaction of 3A with GBF1 interferes with COP-I recruitment, yielding uncoated membranes that cannot participate in secretory pathway trafficking (16, 22–24).

The 3A proteins of PV, EV-A71, and CVB3 also recruit phosphatidylinositol-4-kinase type III β (PI4KIIIβ), which is a critical host factor for viral RNA replication, to the sites of RNA replication (22, 25). PI4KIIIβ, another downstream effector of Arf1, catalyzes the synthesis of phosphatidylinositol-4-phosphate (PI4P) lipids in Golgi membranes. These PI4P lipids not only are precursors in the classical inositol cycle that is important in Ca^2+^ signaling cascades but also have recently been demonstrated to exhibit other functions, such as promoting biogenesis and fusion of transport vesicles in the secretory pathway (26, 27). In enterovirus-infected cells, PI4KIIIβ is actively recruited to replication sites, generating a microenvironment that is rich in PI4P lipids. This environment may attract the RNA-dependent RNA polymerase 3D^pol^ to the replication complex, because 3D^pol^ specifically binds PI4P over other cellular lipids *in vitro* (22). Finally, 3D^pol^ promotes the synthesis of viral RNA.

Aichi virus (a member of the genus *Kobuvirus* of the *Picornaviridae* family) was recently shown to recruit PI4KIIIβ by interacting with ACBD3 (acyl coenzyme A [acyl-CoA]-binding protein 3), which is a newly identified interaction partner of PI4KIIIβ (28, 29). ACBD3 is an essential host factor for pan-enterovirus replication (30). Interestingly, PV replication has also been demonstrated to be inhibited by ACBD3 knockdown (29). Extensive affinity purification and proteomics investigations have established the relationship between 3A proteins of *Kobuvirus* and *Enterovirus* with ACBD3 and PI4KIIIβ. These proteins form a PI4KIIIβ/ACBD3/3A protein complex for viral replication (29).

The inhibition of GBF1 or Arf1 either by pharmacological means or by small interfering RNA (siRNA)-mediated depletion has been demonstrated not to influence the capacity of the CVB3 3A protein to recruit PI4KIIIβ (31). Studies have established that, despite the critical role of GBF1 and PI4KIIIβ in replication, enteroviruses can become resistant to inhibitors that target these factors, suggesting that other pathways or factors are involved in recruiting replication organelles for viral RNA synthesis (32). In this investigation, we attempted to determine the interactions of 3A protein of EV-A71 with other viral and cellular factors during virus replication. We generated recombinant EV-A71 that harbors the FLAG tag in the N-terminal region of 3A protein. We used FLAG antibody to capture 3A protein interactions via immunoprecipitation and utilized proteomics approaches to identify interacting proteins. We focused on one protein, secretory carrier membrane protein 3 (SCAMP3), and examined the functional consequences of its association with 3A. Our results demonstrate that SCAMP3 is a novel host factor that associates with enterovirus 3A protein, PI4KIIIβ, and PI4P to form a replication complex and positively regulates viral replication.

## RESULTS

### Identification of host and viral proteins interacting with EV-A71 3A proteins.

**FIG 1:**
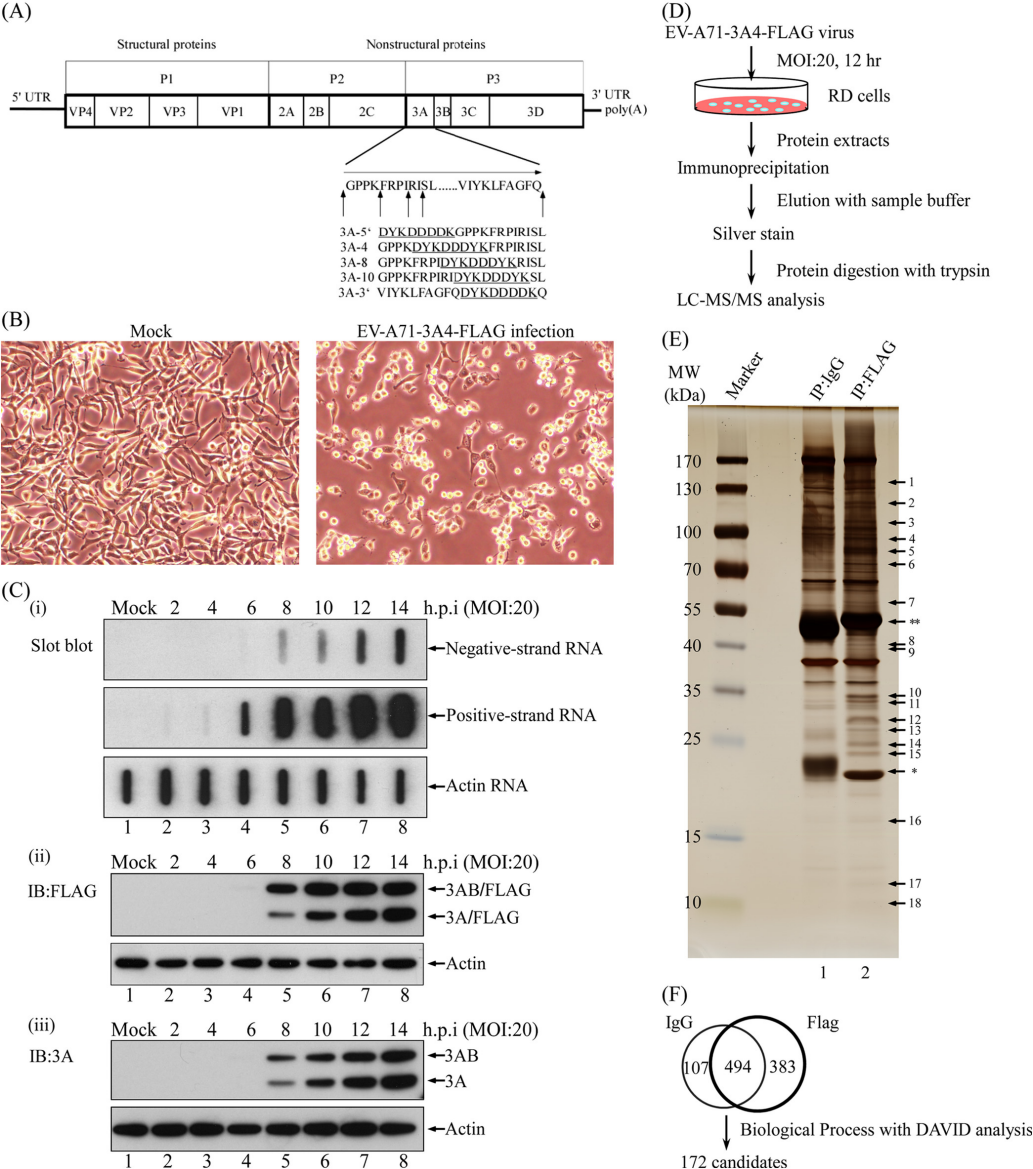
Interaction of proteins with tagged protein 3A from EV-A71-infected cells. (A) Genome organization of EV-A71 and 3A protein. Arrows indicate sites of insertion. Amino acids that encode the FLAG tag are underlined. (B) Cytopathic effect of EV-A71-3A4-FLAG recombinant virus at an MOI of 20 at 12 h postinfection (magnification, 200x). (C) Effects of EV-A71-3A4-FLAG recombinant virus on viral RNA replication and viral protein synthesis. RD cells were infected with EV-A71-3A4-FLAG virus at an MOI of 20 or were mock infected. (i) Cells were collected to detect positive-strand viral RNA or negative-strand viral RNA by slot blot at different times following viral infection. (ii and iii) Cells were collected to detect viral protein levels using Western blotting at various times points after infection. (D) Experimental strategy for the immunoaffinity assay. RD cells were infected with EV-A71-3A4-FLAG recombinant virus at an MOI of 20. Infected-cell lysates were immunoprecipitated with control IgG or anti-FLAG antibodies, washed, and eluted with sample buffer. The sample that contained eluted proteins was then boiled, subjected to 8 to 16% SDS-PAGE, and visualized by silver staining. Protein bands were excised and identified by in-gel trypsin digestion and analyzed by LC-MS/MS. (E) RD cells were infected with EV-A71-3A4-FLAG recombinant virus at an MOI of 20 for 12 h. Immunoaffinity purification via using IgG or anti-FLAG antibody with infected-cell lysates was performed. Protein eluates from the affinity beads were resolved by 8 to 16% SDS-PAGE, and the gel was silver stained. *, light chain; **, heavy chain. (F) From LC-MS/MS data, 1,576 proteins were identified. Following analysis using Proteome Discoverer Daemon 1.4 software, 984 proteins were selected and separated into two groups. The candidate proteins that were associated only with the FLAG group were selected for DAVID analysis, yielding 172 candidates, including GBF1 and ACBD3, which have been reported to be proteins that interact with 3A.

The viral protein 3A sequence performs various functions during enterovirus replication, as both a 3A cleavage product and a precursor such as 3AB. To identify sites in protein 3A that are suitable for the insertion of specific polypeptide tags that do not prevent virus replication, various recombinant EV-A71-3A-FLAG infectious clones were constructed (Fig. 1A). Selected, single-clone EV-A71-3A4-FLAG recombinant viruses were obtained by plaque purification and amplified for further study. Figure 1B shows the cytopathic effect (CPE) of an EV-A71-3A4-FLAG recombinant virus that was rescued following *in vitro* transcription and transfection of RNA derived from an infectious clone.

To identify host factors that interact with 3A protein, viral RNA and protein levels during infection by EV-A71-3A4-FLAG recombinant virus were examined. Twelve hours postinfection with a multiplicity of infection (MOI) of 20 of virus, at the point of maximal recombinant viral RNA replication, both viral RNA and viral protein levels rapidly increased (Fig. 1C). Twelve-hour-infected cell lysates were collected to perform a FLAG immunoprecipitation assay. 3A-FLAG-interacting proteins were pulled down and detected using one-dimensional, sodium dodecyl sulfate-polyacrylamide gel electrophoresis (1D SDS-PAGE). Potential interacting proteins were identified by liquid chromatography-tandem mass spectrometry (LC-MS/MS) analysis (Fig. 1D). From the results in Fig. 1E, 18 bands of the anti-FLAG group (Fig. 1E, lane 2) and the bands of the control IgG group (Fig. 1E, lane 1) were cut and subjected to in-gel digestion and LC-MS/MS analysis.

From the LC-MS/MS data, approximately 1,576 proteins were identified. Following an analysis performed using Proteome Discoverer Daemon 1.4 software, 984 of these proteins were divided into two groups. Candidate proteins that were associated only with the FLAG group underwent DAVID analysis (*P* value < 0.01, Benjamini value < 0.01) (Fig. 1F). The 172 candidates are shown in Table 1 with their accession numbers obtained from the NCBI protein database. These 172 candidates included GBF1 and ACBD3, which are reportedly associated with viral 3A protein and involved in viral replication. Not only cellular proteins but also the viral proteins 2C, 3A, 3C^pro^, and 3D^pol^ were identified in the experiment.

**TABLE 1:** List of 172 identified proteins in LC-MS/MS measurements

UniProt accession no.	Gene symbol	Protein name^*a*^	MW (kDa)	Protein score	No. of peptides		No. of matched spectra	% coverage
					Unique	Identified		
Q9NRG9	*AAAS*	Aladin	59.5	382.7	4	4	8	8.97
Q9H845	*ACAD9*	Acyl-CoA dehydrogenase family member 9, mitochondrial	68.7	110.6	2	2	2	4.51
Q9H3P7	*ACBD3*	Golgi resident protein GCP60	60.6	189.2	2	2	4	5.30
Q16186	*ADRM1*	Proteasomal ubiquitin receptor ADRM1	42.1	48.2	2	2	3	6.63
Q9NUQ2	*AGPAT5*	1-Acyl-*sn*-glycerol-3-phosphate acyltransferase epsilon	42.0	115.0	3	3	4	9.07
O00116	*AGPS*	Alkyl-dihydroxyacetone-phosphate synthase, peroxisomal	72.9	539.6	9	9	12	17.02
P30837	*ALDH1B1*	Aldehyde dehydrogenase X, mitochondrial	57.2	480.8	7	7	12	16.83
Q9BT22	*ALG1*	Chitobiosyldiphosphodolichol beta-mannosyltransferase	52.5	266.1	8	8	10	22.20
Q6ICH7	*ASPHD2*	Aspartate beta-hydroxylase domain-containing protein 2	41.7	70.8	2	2	2	6.50
Q8NHH9	*ATL2*	Atlastin-2	66.2	96.4	4	4	4	7.72
Q07812	*BAX*	Apoptosis regulator BAX	21.2	268.7	5	5	8	30.73
Q07817	*BCL2L1*	Bcl-2-like protein 1	26.0	86.6	3	3	4	12.02
Q9Y276	*BCS1L*	Mitochondrial chaperone BCS1	47.5	173.2	5	5	6	14.08
O15155	*BET1*	BET1 homolog	13.3	219.2	2	2	4	24.58
P55957	*BID*	BH3-interacting domain death agonist	22.0	235.0	3	3	4	20.00
Q9UKR5	*C14orf1*	Probable ergosterol biosynthetic protein 28	15.9	70.2	3	3	4	19.29
P21926	*CD9*	CD9 antigen	25.4	85.0	2	2	4	7.46
O76031	*CLPX*	ATP-dependent Clp protease ATP-binding subunit clpX-like, mitochondrial	69.2	364.4	6	6	9	11.06
P09496	*CLTA*	Clathrin light chain A	27.1	103.4	2	2	3	7.26
P09497	*CLTB*	Clathrin light chain B	25.2	84.4	2	2	3	8.30
Q92905	*COPS5*	COP9 signalosome complex subunit 5	37.6	314.3	5	5	8	17.96
Q9Y2Z9	*COQ6*	Ubiquinone biosynthesis monooxygenase COQ6	50.8	328.5	7	7	12	15.81
Q7KZN9	*COX15*	Cytochrome *c* oxidase assembly protein COX15 homolog	46.0	35.5	2	2	2	4.63
Q13363	*CTBP1*	C-terminal-binding protein 1	47.5	95.6	3	3	3	6.14
Q13618	*CUL3*	Cullin-3	88.9	254.3	5	5	8	6.38
Q92499	*DDX1*	ATP-dependent RNA helicase DDX1	82.4	107.5	3	3	4	5.27
Q9BUN8	*DERL1*	Derlin-1	28.8	198.0	2	2	4	9.96
O75907	*DGAT1*	Diacylglycerol *O*-acyltransferase 1	55.2	167.1	2	2	3	5.94
Q15392	*DHCR24*	24-Dehydrocholesterol reductase	60.1	40.1	3	3	5	4.26
Q9UBM7	*DHCR7*	7-Dehydrocholesterol reductase	54.5	181.6	2	2	3	5.05
Q9Y394	*DHRS7*	Dehydrogenase/reductase SDR family member 7	38.3	123.1	2	2	2	7.08
Q6IAN0	*DHRS7B*	Dehydrogenase/reductase SDR family member 7B	35.1	387.9	5	5	7	20.00
Q96DA6	*DNAJC19*	Mitochondrial import inner membrane translocase subunit TIM14	12.5	215.6	2	2	3	28.45
Q9H3H5	*DPAGT1*	UDP-*N*-acetylglucosamine-dolichyl-phosphate *N*-acetylglucosamine phosphotransferase	46.1	169.9	3	3	6	6.62
O60762	*DPM1*	Dolichol-phosphate mannosyltransferase	29.6	150.2	2	2	3	12.69
Q5JPH6	*EARS2*	Probable glutamyl-tRNA synthetase, mitochondrial	58.7	41.6	2	2	2	4.21
Q05639	*EEF1A2*	Elongation factor 1-alpha 2	50.4	854.2	4	12	27	37.37
P24534	*EEF1B2*	Elongation factor 1-beta	24.7	364.6	5	5	9	31.56
P55884	*EIF3B*	Eukaryotic translation initiation factor 3 subunit B	92.4	579.0	10	10	16	14.99
Q99613	*EIF3C*	Eukaryotic translation initiation factor 3 subunit C	105.3	65.7	2	2	2	2.19
Q9Y262	*EIF3L*	Eukaryotic translation initiation factor 3 subunit L	66.7	73.0	2	2	2	3.37
Q04637	*EIF4G1*	Eukaryotic translation initiation factor 4 gamma 1	175.4	91.4	3	3	4	2.31
Q96RQ1	*ERGIC2*	Endoplasmic reticulum-Golgi intermediate compartment protein 2	42.5	65.1	2	2	2	7.96
Q9Y285	*FARSA*	Phenylalanyl-tRNA synthetase alpha chain	57.5	382.4	4	4	8	9.06
P49327	*FASN*	Fatty acid synthase	273.3	109.4	2	2	2	1.12
P21333	*FLNA*	Filamin-A	280.6	115.6	4	4	4	1.66
P41250	*GARS*	Glycyl-tRNA synthetase	83.1	126.1	6	6	8	8.66
Q92538	*GBF1*	Golgi-specific brefeldin A-resistance guanine nucleotide exchange factor 1	206.3	1,992.4	30	30	55	19.10
O15228	*GNPAT*	Dihydroxyacetone phosphate acyltransferase	77.1	130.5	3	3	4	4.85
Q8N335	*GPD1L*	Glycerol-3-phosphate dehydrogenase 1-like protein	38.4	104.0	4	4	4	11.68
Q92522	*H1FX*	Histone H1x	22.5	245.7	4	4	6	22.54
P68431	*HIST1H3A*	Histone H3.1	15.4	85.2	3	3	4	16.91
P09601	*HMOX1*	Heme oxygenase 1	32.8	180.0	5	5	7	24.65
P30519	*HMOX2*	Heme oxygenase 2	36.0	111.8	4	4	4	16.77
P56937	*HSD17B7*	3-Keto-steroid reductase	38.2	367.3	9	9	14	34.31
P41252	*IARS*	Isoleucyl-tRNA synthetase, cytoplasmic	144.4	285.5	9	9	11	8.72
P11717	*IGF2R*	Cation-independent mannose-6-phosphate receptor	274.1	57.1	2	2	2	0.84
Q13418	*ILK*	Integrin-linked protein kinase	51.4	53.2	2	2	2	3.98
Q96P70	*IPO9*	Importin-9	115.9	195.3	3	3	4	4.13
P06756	*ITGAV*	Integrin alpha-V	116.0	60.9	3	3	4	2.48
O43731	*KDELR3*	ER lumen protein retaining receptor 3	25.0	195.6	3	3	4	14.49
Q92945	*KHSRP*	Far upstream element-binding protein 2	73.1	150.5	4	4	5	6.48
P52292	*KPNA2*	Importin subunit alpha-2	57.8	254.1	3	3	6	9.64
Q15031	*LARS2*	Probable leucyl-tRNA synthetase, mitochondrial	101.9	334.1	9	9	12	11.07
Q9H0V9	*LMAN2L*	VIP36-like protein	39.7	288.4	6	6	11	22.70
Q8NF37	*LPCAT1*	Lysophosphatidylcholine acyltransferase 1	59.1	209.2	2	2	3	6.74
Q02750	*MAP2K1*	Dual specificity mitogen-activated protein kinase kinase 1	43.4	88.5	2	2	3	5.09
P56192	*MARS*	Methionyl-tRNA synthetase, cytoplasmic	101.1	207.0	5	5	6	6.33
P49736	*MCM2*	DNA replication licensing factor MCM2	101.8	59.1	2	2	2	2.54
Q96HR3	*MED30*	Mediator of RNA polymerase II transcription subunit 30	20.3	124.4	3	3	3	16.85
Q8IWA4	*MFN1*	Mitofusin-1	84.0	183.3	4	4	4	7.29
Q9BYD6	*MRPL1*	39S Ribosomal protein L1, mitochondrial	36.9	244.5	3	3	5	17.54
Q9P015	*MRPL15*	39S Ribosomal protein L15, mitochondrial	33.4	97.3	2	2	3	8.11
Q5T653	*MRPL2*	39S Ribosomal protein L2, mitochondrial	33.3	200.5	3	3	4	15.41
Q96A35	*MRPL24*	39S Ribosomal protein L24, mitochondrial	24.9	241.8	3	3	5	16.67
Q9P0M9	*MRPL27*	39S Ribosomal protein L27, mitochondrial	16.1	69.2	2	2	3	16.89
Q13084	*MRPL28*	39S Ribosomal protein L28, mitochondrial	30.1	80.3	2	2	3	12.89
Q9BYD3	*MRPL4*	39S Ribosomal protein L4, mitochondrial	34.9	201.9	6	6	8	25.72
Q8IXM3	*MRPL41*	39S Ribosomal protein L41, mitochondrial	15.4	74.3	3	3	4	26.28
Q9NZJ7	*MTCH1*	Mitochondrial carrier homolog 1	41.5	88.8	3	3	3	8.48
P13995	*MTHFD2*	Bifunctional methylenetetrahydrofolate dehydrogenase/cyclohydrolase, mitochondrial	37.9	307.2	5	5	7	22.00
Q13505	*MTX1*	Metaxin-1	51.4	360.0	7	7	12	19.10
P54920	*NAPA*	Alpha-soluble NSF attachment protein	33.2	628.4	9	9	16	41.36
O43678	*NDUFA2*	NADH dehydrogenase (ubiquinone) 1 alpha subcomplex subunit 2	10.9	167.5	2	2	3	30.30
O95182	*NDUFA7*	NADH dehydrogenase (ubiquinone) 1 alpha subcomplex subunit 7	12.5	107.9	4	4	5	39.82
Q9Y375	*NDUFAF1*	Complex I intermediate-associated protein 30	37.7	138.5	3	3	7	11.62
O75380	*NDUFS6*	NADH dehydrogenase (ubiquinone) iron-sulfur protein 6, mitochondrial	13.7	117.9	2	2	3	21.77
P49821	*NDUFV1*	NADH dehydrogenase (ubiquinone) flavoprotein 1, mitochondrial	50.8	259.1	6	6	8	13.36
P01111	*NRAS*	GTPase NRas	21.2	636.1	4	6	18	39.15
Q8WUM0	*NUP133*	Nuclear pore complex protein Nup133	128.9	285.9	7	7	12	6.83
O75694	*NUP155*	Nuclear pore complex protein Nup155	155.1	293.5	7	7	11	5.25
Q12769	*NUP160*	Nuclear pore complex protein Nup160	162.0	67.9	2	2	2	1.67
Q92621	*NUP205*	Nuclear pore complex protein Nup205	227.8	214.9	3	3	4	2.14
Q8NFH3	*NUP43*	Nucleoporin Nup43	42.1	73.9	2	2	3	6.32
Q7Z3B4	*NUP54*	Nucleoporin p54	55.4	70.0	3	3	3	7.50
O60313	*OPA1*	Dynamin-like 120-kDa protein, mitochondrial	111.6	676.3	12	12	17	15.31
P08559	*PDHA1*	Pyruvate dehydrogenase El component subunit alpha, somatic form, mitochondrial	43.3	165.3	4	4	6	10.00
P11177	*PDHB*	Pyruvate dehydrogenase E1 component subunit beta, mitochondrial	39.2	353.9	3	3	6	11.98
O00623	*PEX12*	Peroxisome assembly protein 12	40.8	74.2	2	2	3	5.85
P40855	*PEX19*	Peroxisomal biogenesis factor 19	32.8	347.1	4	4	6	23.41
Q01813	*PFKP*	6-Phosphofructokinase type C	85.5	134.4	3	3	3	5.36
O43175	*PHGDH*	d-3-Phosphoglycerate dehydrogenase	56.6	775.5	11	11	17	26.45
Q969N2	*PIGT*	GPI transamidase component PIG-T	65.7	46.0	4	4	4	5.19
Q9UG56	*PISD*	Phosphatidylserine decarboxylase proenzyme	46.5	224.6	4	4	7	10.54
Q13362	*PPP2R5C*	Serine/threonine-protein phosphatase 2A 56-kDa regulatory subunit gamma isoform	61.0	92.1	3	3	4	8.02
P50897	*PPT1*	Palmitoyl-protein thioesterase 1	34.2	281.7	5	5	7	31.37
Q9HCU5	*PREB*	Prolactin regulatory element-binding protein	45.4	424.1	7	7	11	26.14
P54619	*PRKAG1*	5′-AMP-activated protein kinase subunit gamma-1	37.6	55.0	2	2	2	6.34
P04156	*PRNP*	Major prion protein	27.6	87.4	2	2	2	7.91
O94906	*PRPF6*	Pre-mRNA-processing factor 6	106.9	47.1	2	2	2	1.91
P62195	*PSMC5*	26S Protease regulatory subunit 8	45.6	136.6	3	3	4	9.11
P62333	*PSMC6*	26S Protease regulatory subunit S10B	44.1	121.0	3	3	3	9.77
Q99460	*PSMD1*	26S Proteasome non-ATPase regulatory subunit 1	105.8	138.1	3	3	3	4.62
O00231	*PSMD11*	26S Proteasome non-ATPase regulatory subunit 11	47.4	138.0	2	2	3	5.45
P51665	*PSMD7*	26S Proteasome non-ATPase regulatory subunit 7	37.0	165.5	2	2	3	7.41
P61289	*PSME3*	Proteasome activator complex subunit 3	29.5	147.2	2	2	2	10.24
P53801	*PTTG1IP*	Pituitary tumor-transforming gene 1 protein-interacting protein	20.3	73.8	2	2	2	13.89
Q53H96	*PYCRL*	Pyrroline-5-carboxylate reductase 3	28.6	171.1	3	3	5	17.15
P61106	*RAB14*	Ras-related protein Rab-14	23.9	213.1	3	3	4	20.47
Q9ULC3	*RAB23*	Ras-related protein Rab-23	26.6	71.9	2	2	3	8.02
P51149	*RAB7A*	Ras-related protein Rab-7a	23.5	155.8	3	3	4	16.91
P43487	*RANBP1*	Ran-specific GTPase-activating protein	23.3	45.7	2	2	2	9.95
P49792	*RANBP2*	E3 SUMO-protein ligase RanBP2	358.0	739.7	11	11	20	4.19
P35241	*RDX*	Radixin	68.5	53.7	3	3	4	4.12
Q6NUM9	*RETSAT*	All-*trans*-retinol 13,14-reductase	66.8	295.1	4	4	7	7.70
P62266	*RPS23*	40S Ribosomal protein S23	15.8	155.7	2	2	3	15.38
P62241	*RPS8*	40S Ribosomal protein S8	24.2	77.5	2	2	2	11.54
Q9P2E9	*RRBP1*	Ribosome-binding protein 1	152.4	211.5	5	5	5	4.26
P23921	*RRM1*	Ribonucleoside-diphosphate reductase large subunit	90.0	54.1	3	3	3	3.54
O15126	*SCAMP1*	Secretory carrier-associated membrane protein 1	37.9	228.8	3	3	6	13.91
O14828	*SCAMP3*	Secretory carrier-associated membrane protein 3	38.3	262.5	3	3	6	10.37
Q8WTV0	*SCARB1*	Scavenger receptor class B member 1	60.8	100.0	4	4	5	6.88
O00767	*SCD*	Acyl-CoA desaturase	41.5	142.8	2	2	3	6.96
Q86SK9	*SCD5*	Stearoyl-CoA desaturase 5	37.6	176.7	4	4	7	15.76
Q9NVU7	*SDAD1*	Protein SDA1 homolog	79.8	100.1	2	2	2	3.78
P21912	*SDHB*	Succinate dehydrogenase (ubiquinone) iron-sulfur subunit, mitochondrial	31.6	257.2	6	6	10	22.14
P60468	*SEC61B*	Protein transport protein Sec61 subunit beta	10.0	70.5	2	2	3	26.04
Q96EE3	*SEH1L*	Nucleoporin SEH1	39.6	102.1	3	3	3	11.94
Q13247	*SFRS6*	Splicing factor, arginine/serine-rich 6	39.6	60.7	2	2	2	4.65
Q9Y371	*SH3GLB1*	Endophilin-B1	40.8	73.3	3	3	3	8.49
P53007	*SLC25A1*	Tricarboxylate transport protein, mitochondrial	34.0	496.9	8	8	18	30.87
Q9UBX3	*SLC25A10*	Mitochondrial dicarboxylate carrier	31.3	297.4	5	5	8	23.69
O95258	*SLC25A14*	Brain mitochondrial carrier protein 1	36.2	101.5	2	2	2	8.62
Q9Y619	*SLC25A15*	Mitochondrial ornithine transporter 1	32.7	138.8	4	4	6	14.95
O43772	*SLC25A20*	Mitochondrial carnitine/acylcarnitine carrier protein	32.9	194.7	8	8	12	23.59
P12235	*SLC25A4*	ADP/ATP translocase 1	33.0	1,909.2	5	15	65	51.68
Q99808	*SLC29A1*	Equilibrative nucleoside transporter 1	50.2	95.7	3	3	5	7.46
P62318	*SNRPD3*	Small nuclear ribonucleoprotein Sm D3	13.9	66.1	2	2	2	15.08
Q9UNH7	*SNX6*	Sorting nexin-6	46.6	161.1	6	6	8	14.29
Q99523	*SORT1*	Sortilin	92.0	362.6	7	7	11	9.87
O76094	*SRP72*	Signal recognition particle 72-kDa protein	74.6	61.5	3	3	4	7.75
P43307	*SSR1*	Translocon-associated protein subunit alpha	32.2	307.9	3	3	6	11.89
Q9UNL2	*SSR3*	Translocon-associated protein subunit gamma	21.1	89.8	4	4	6	12.43
O14662	*STX16*	Syntaxin-16	37.0	330.1	5	5	8	18.77
P32856	*STX2*	Syntaxin-2	33.3	68.0	2	2	3	6.60
Q9P2R7	*SUCLA2*	Succinyl-CoA ligase (ADP-forming) subunit beta, mitochondrial	50.3	76.3	2	2	2	4.10
P53597	*SUCLG1*	Succinyl-CoA ligase (GDP-forming) subunit alpha, mitochondrial	36.2	223.8	4	4	8	15.03
Q86TM6	*SYVN1*	E3 ubiquitin-protein ligase synoviolin	67.6	55.6	2	2	3	2.59
P17987	*TCP1*	T-complex protein 1 subunit alpha	60.3	41.3	2	2	2	3.96
Q9BTX1	*TMEM48*	Nucleoporin NDC1	76.3	319.3	6	6	11	10.53
O14787	*TNPO2*	Transportin-2	101.3	206.6	2	2	4	2.45
Q9Y5L0	*TNPO3*	Transportin-3	104.1	95.6	2	2	2	3.25
P43897	*TSFM*	Elongation factor Ts, mitochondrial	35.4	65.6	2	2	2	6.46
Q99816	*TSG101*	Tumor susceptibility gene 101 protein	43.9	70.1	2	2	2	5.13
P68371	*TUBB2C*	Tubulin beta-2C chain	49.8	1,577.6	2	14	38	47.87
Q9BUF5	*TUBB6*	Tubulin beta-6 chain	49.8	1,040.8	3	10	27	30.27
P07919	*UQCRH*	Cytochrome *b-c*1 complex subunit 6, mitochondrial	10.7	52.2	2	2	2	27.47
Q3ZAQ7	*VMA21*	Vacuolar ATPase assembly integral membrane protein VMA21	11.3	66.3	2	2	2	21.78
Q9NRW7	*VPS45*	Vacuolar protein sorting-associated protein 45	65.0	81.4	2	2	3	4.39
Q9UIA9	*XPO7*	Exportin-7	123.8	291.4	11	11	13	10.76
O43592	*XPOT*	Exportin-T	109.9	256.6	4	4	7	5.09
O75844	*ZMPSTE24*	CAAX prenyl protease 1 homolog	54.8	251.6	4	4	7	11.58

a CoA, coenzyme A.

From the DAVID analysis, the cellular biological functions of the aforementioned 172 proteins are related to protein localization, intracellular transport, and membrane organization, among others (Table 2). Based on their biological functions and LC-MS/MS scores, some of the 172 candidate cellular proteins were examined further; one of them, secretory carrier membrane protein 3 (SCAMP3), was chosen specifically since it is a novel 3A-associated protein and participates in protein localization and intracellular transport (Table 2).

**TABLE 2:** Enrichment analysis of biological processes in EV-A71-infected RD cells

Biological process	Identified proteins involved in the process	*P* value	Benjamini value
Intracellular transport	CLTA, CLTB, ATL2, LMAN2L, SSR1, SLC25A20, GBF1, RANBP1, SLC25A1, RANBP2, DNAJC19, SCAMP1, NUP133, SCAMP3, OPA1, STX2, VPS45, IPO9, ERGIC2, FLNA, AAAS, SEC61B, NUP205, RAB14, SORT1, SRP72, KPNA2, BID, DERL1, SNX6, NUP160, MTX1, BET1, NAPA, BCL2L1, PEX19, STX16, NUP54, PEX12, TNPO2, XPOT, MAP2K1, NUP155, PREB, SLC25A14, BAX, SLC25A10, PTTG1IP, XPO7, SLC25A15, SSR3	1.60 × 10^−14^	3.00 × 10^−11^
Protein localization	CLTA, CLTB, TSG101, LMAN2L, SSR1, SEH1L, RAB23, RANBP2, DNAJC19, DHCR24, KDELR3, SCAMP1, NUP133, SCAMP3, STX2, VPS45, IPO9, CLPX, FLNA, SEC61B, NUP205, RAB14, SORT1, SRP72, KPNA2, NUP43, BID, RAB7A, SDAD1, DERL1, SNX6, NUP16 0, MTX1, BET1, NAPA, RDX, PPT1, PEX19, SH3GLB1, STX16, NUP54, PEX12, TNPO2, TNPO3, RRBP1, NUP155, PREB, TMEM48, BAX, PTTG1IP, XPO7, SSR3	2.69 × 10^−10^	1.68 × 10^−7^
Translation	MRPL41, EEF1B2, COPS5, IARS, EIF3C, EIF3B, MRPL15, EIF3L, RPS23, MARS, MRPL2, MRPL1, MRPL4, RRBP1, EEF1A2, GARS, LARS2, RPS8, EARS2, EIF4G1, MRPL24, MRPL28, MRPL27, TSFM, FARSA	4.08 × 10^−7^	1.09 × 10^−4^
Nucleobase, nucleoside, nucleotide, and nucleic acid transport	XPOT, NUP133, SLC25A4, NUP160, NUP155, SLC29A1, TMEM48, SEH1L, NUP205, KHSRP, RANBP2, NUP54, XPO7, NUP43	1.37 × 10^−6^	2.86 × 10^−4^
Macromolecular complex subunit organization	ATL2, PRKAG1, SNRPD3, TUBB2C, H1FX, NDUFAF1, SFRS6, SEH1L, ILK, VMA21, TUBB6, SCARB1, TNPO2, COX15, NUP133, TCP1, MAP2K1, DDX1, PFKP, DPAGT1, IPO9, BCS1L, MCM2, FLNA, PRPF6, ADRM1, MED30, TMEM48, DGAT1, UQCRH, NUP205, BAX, RRM1, HIST1H3A, XPO7, PRNP	8.86 × 10^−6^	1.66 × 10^−3^
Cellular respiration	NDUFS6, SDHB, SLC25A14, NDUFA2, UQCRH, NDUFV1, SUCLG1, NDUFA7, SUCLA2, NDUFAF1, PDHB, COX15	1.03 × 10^−5^	1.76 × 10^−3^
Membrane organization	SCAMP1, BID, RAB7A, CLTA, OPA1, STX2, NAPA, PPT1, BCL2L1, PREB, CD9, NRAS, MFN1, GBF1, SH3GLB1, IGF2R, ITGAV, BAX, MTCH1, GNPAT, SORT1, ZMPSTE24, SCARB1, DHCR24	1.53 × 10^−5^	2.21 × 10^−3^
Mitochondrial transport	BID, SLC25A20, SLC25A14, BAX, SLC25A10, MTX1, SLC25A1, BCL2L1, SLC25A15, DNAJC19	2.14 × 10^−5^	2.86 × 10^−3^
Nucleocytoplasmic transport	XPOT, NUP133, NUP160, IPO9, NUP155, AAAS, SEC61B, NUP205, PTTG1IP, RANBP2, NUP54, TNPO2, XPO7, KPNA2	4.73 × 10^−5^	4.65 × 10^−3^
Proteasomal protein catabolic process	CUL3, PSMC6, SEC61B, DERL1, PSMC5, SYVN1, PSMD11, PPP2R5C, PSMD1, PSME3, PSMD7	9.20 × 10^−5^	7.81 × 10^−3^
Oxidation reduction	PYCRL, NDUFAF1, PDHB, GPD1L, HMOX2, MTHFD2, NDUFS6, HMOX1, DHCR7, FASN, PDHA1, SCD5, ACAD9, HSD17B7, DHCR24, COX15, CTBP1, NDUFA2, SCD, NDUFA7, DHRS7B, COQ6, DHRS7, SDHB, UQCRH, ALDH1B1, ASPHD2, NDUFV1, RRM1, PHGDH, RETSAT	9. 82 × 10^−5^	7.64 × 10^−3^
Lipid biosynthetic process	ALG1, PRKAG1, SCD, PIGT, DPAGT1, PISD, C14ORF1, ACBD3, DGAT1, AGPAT5, AGPS, LPCAT1, SH3GLB1, DHCR7, FASN, DPM1, SCARB1, SCD5, HSD17B7, DHCR24	1.20 × 10^−4^	8.97 × 10^−3^

### SCAMP3 associates with 3A protein in EV-A71 infection.

**FIG 2:**
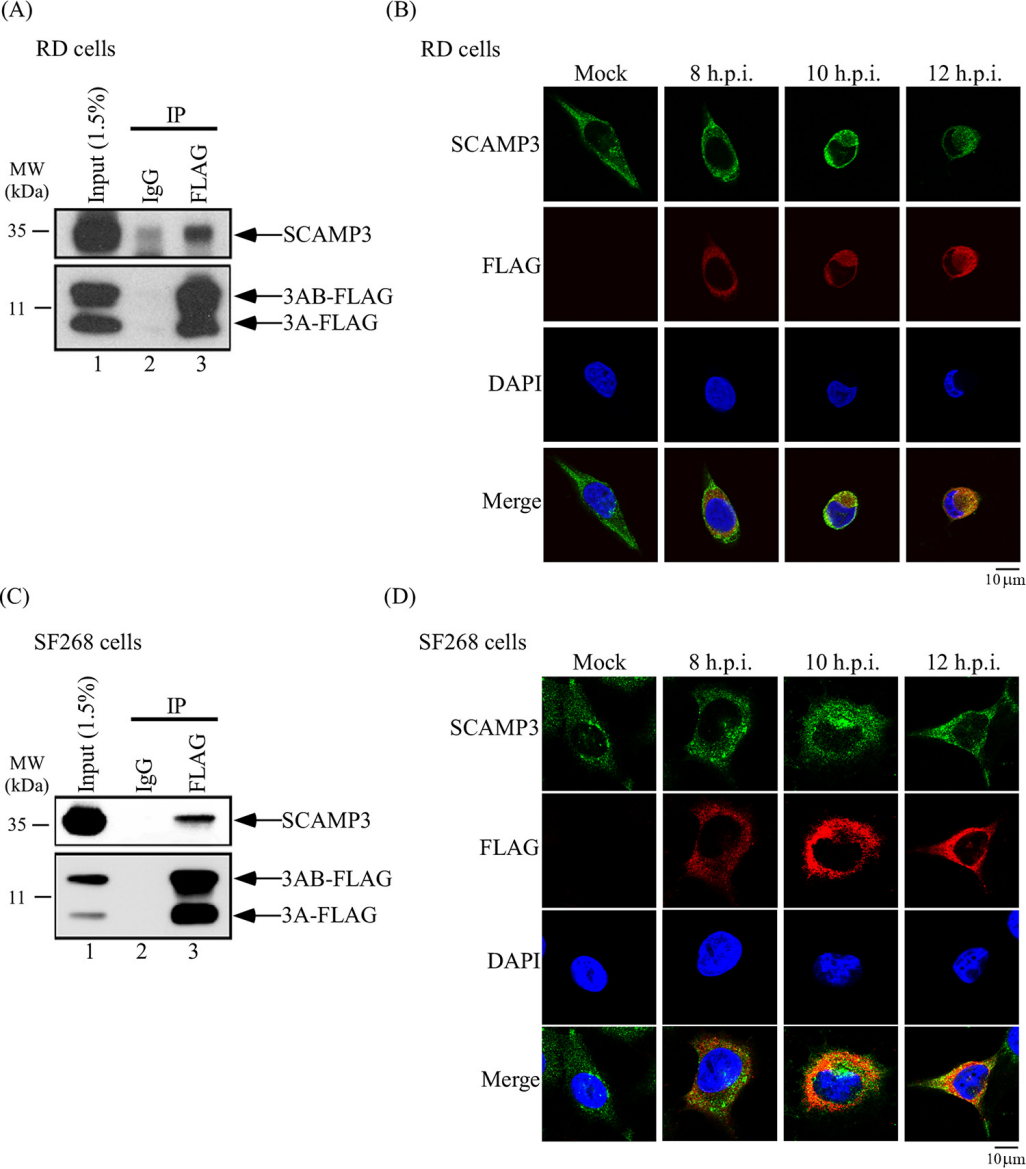
3A protein interacts and colocalizes with SCAMP3 in EV-A71-infected cells. (A) A co-immunoprecipitation assay was carried out. RD cells were infected with EV-A71-3A4-FLAG recombinant virus at an MOI of 20 for 12 h. Infected-cell lysates were immunoprecipitated with control IgG or anti-FLAG antibodies, and proteins that bound to the resin were analyzed by 12% SDS-PAGE, followed by immunoblotting with anti-SCAMP3 antibody. (B) Colocalization of SCAMP3 with 3A protein. RD cells were mock infected or infected with EV-A71 at an MOI of 20. At different time points postinfection, cells were fixed with formaldehyde, washed, and immunostained with antibody against SCAMP3 or 3A-FLAG. DAPI was used to stain the nucleus. Images were captured by confocal laser scanning microscopy. Scale bar, 10 µm. (C) A co-immunoprecipitation assay was carried out. SF268 cells were infected with EV-A71-3A4-FLAG recombinant virus at an MOI of 20 for 12 h. Infected-cell lysates were immunoprecipitated with control IgG or anti-FLAG antibodies, and proteins that bound to the resin were analyzed by 12% SDS-PAGE, followed by immunoblotting with anti-SCAMP3 antibody. (D) Colocalization of SCAMP3 with 3A protein. SF268 cells were mock infected or infected with EV-A71 at an MOI of 20. At different time points postinfection, cells were fixed with formaldehyde, washed, and immunostained with antibody against SCAMP3 or 3A-FLAG. DAPI was used to stain the nucleus. Images were captured by confocal laser scanning microscopy. Scale bar, 10 μm.

The interaction of SCAMP3 and 3A was further verified using lysates of EV-A71-infected RD cells by co-immunoprecipitation (co-IP) and Western blot assays with antibodies against endogenous SCAMP3 protein and FLAG, respectively. The result suggests that endogenous SCAMP3 interacts with EV-A71 3A and 3AB at 12 h postinfection (Fig. 2A). To determine whether these interactions also occur in another cell type, co-IP was performed with SF268 cell lysates. As shown in Fig. 2C, SCAMP3 protein interacted with EV-A71 3A and 3AB in SF268 cells at 12 h postinfection. These results indicate that the interactions are not specific to cell type.

To characterize further the cellular localization of SCAMP3 in EV-A71-infected cells, their subcellular localization after EV-A71 infection was compared with that after mock infection using fluorescence microscopy. The localization of 3A-FLAG and SCAMP3 in RD cells, following a time course of EV-A71 infection, was studied using anti-FLAG (red color) and anti-SCAMP3 (green color) antibodies in an immunofluorescence assay (IFA) by confocal microscopy (Fig. 2). The images revealed that 3A protein was colocalized with SCAMP3 at different time points postinfection (Fig. 2B). In EV-A71-infected SF268 cells, 3A protein also colocalized with SCAMP3 (Fig. 2D).

### Knockdown or knockout of SCAMP3 decreases EV-A71 replication.

**FIG 3:**
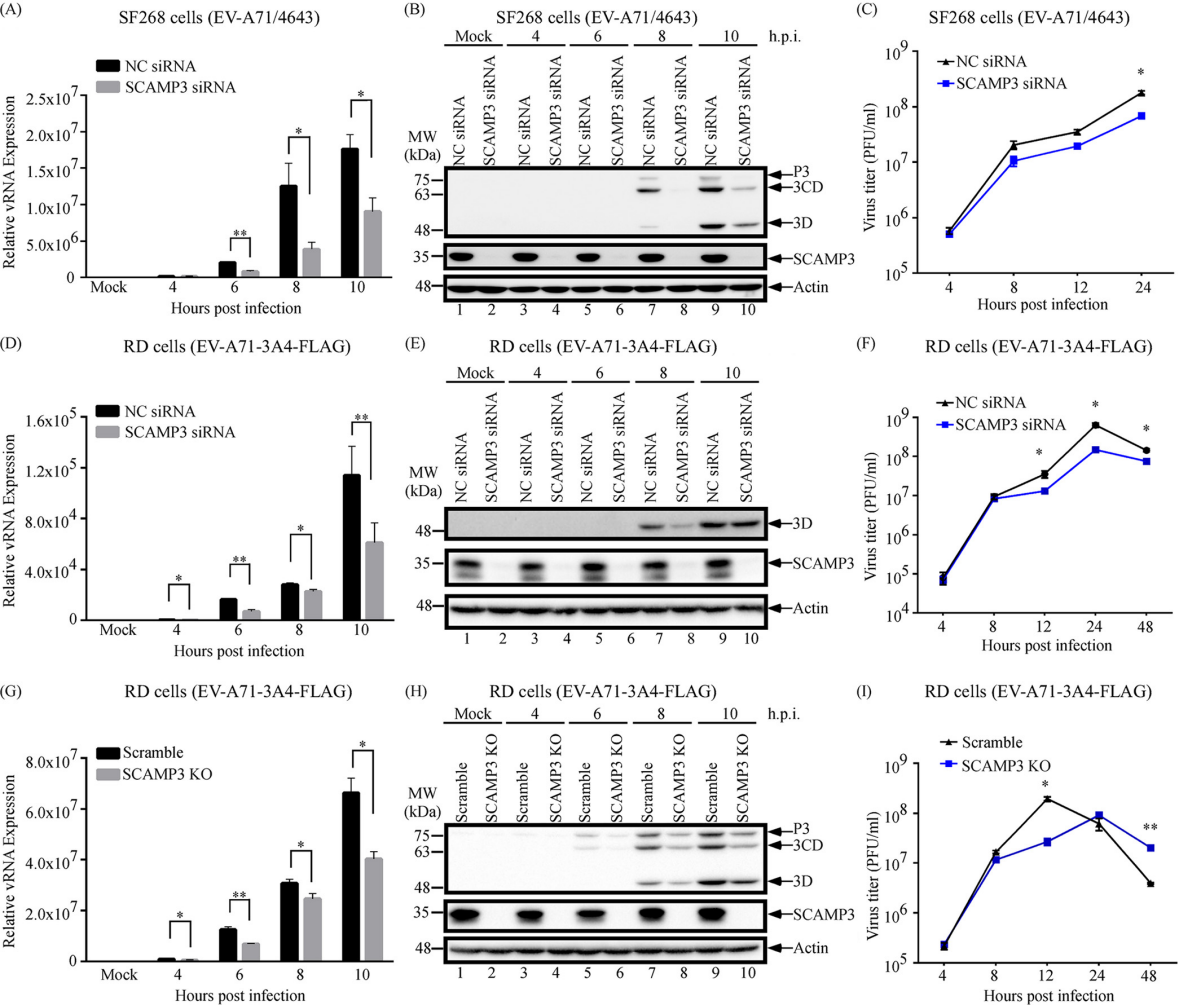
Effects of SCAMP3 knockdown or knockout on EV-A71 replication. (A) Effect of SCAMP3 knockdown on EV-A71/4643 RNA levels. SF268 cells were transfected with NC siRNA or siRNA against SCAMP3. Cells were mock infected or infected with EV-A71/4643 at an MOI of 10 2 days after transfection. Total RNA was extracted and viral RNA levels were determined by quantitative RT-qPCR. (B) Effect of SCAMP3 knockdown on EV-A71/4643 viral 3D, 3CD, and P3 protein levels. SF268 cells were transfected with NC siRNA or siRNA against SCAMP3. Cells were mock infected or infected with EV-A71/4643 at an MOI of 10 2 days after transfection. Total cell lysates were examined by Western blotting. (C) Effect of SCAMP3 knockdown on EV-A71/4643 viral growth. SF268 cells were transfected with NC siRNA or siRNA against SCAMP3. Cells were mock infected or infected with EV-A71 at an MOI of 10 2 days after transfection. Viruses were harvested at different time points postinfection and assayed by plaque formation with RD cells. (D) Effect of SCAMP3 knockdown on EV-A71-3A4-FLAG RNA levels. RD cells were transfected with NC siRNA or siRNA against SCAMP3. Cells were mock infected or infected with EV-A71-3A4-FLAG at an MOI of 20 2 days after transfection. Total RNA was extracted and viral RNA levels were determined by RT-qPCR. (E) Effect of SCAMP3 knockdown on EV-A71-3A4-FLAG viral 3D protein levels. RD cells were transfected with NC siRNA or siRNA against SCAMP3. Cells were mock infected or infected with EV-A71-3A4-FLAG at an MOI of 20 2 days after transfection. Total cell lysates were examined by Western blotting. (F) Effect of SCAMP3 knockdown on EV-A71-3A4-FLAG viral growth. RD cells were transfected with NC siRNA or siRNA against SCAMP3. Cells were mock infected or infected with EV-A71-3A4-FLAG at an MOI of 20 2 days after transfection. Viruses were harvested at different time points postinfection and assayed by plaque formation with RD cells. (G) Effect of SCAMP3 knockout on EV-A71 RNA levels. Scramble or SCAMP3 KO RD cells were mock infected or infected with EV-A71-3A4-FLAG at an MOI of 20. Total RNA was extracted and viral RNA levels were determined by RT-qPCR. (H) Effect of SCAMP3 knockout on EV-A71-3A4-FLAG viral 3D, 3CD, and P3 protein levels. Scramble or SCAMP3 KO RD Cells were mock infected or infected with EV-A71-3A4-FLAG at an MOI of 20. Total cell lysates were examined by Western blotting. (I) Effect of SCAMP3 knockout on EV-A71-3A4-FLAG viral growth. Scramble or SCAMP3 KO RD Cells were mock infected or infected with EV-A71-3A4-FLAG at an MOI of 20. Viruses were harvested at different time points postinfection and assayed by plaque formation with RD cells.

Effects of SCAMP3 knockdown on EV-A71 viral RNA synthesis, translation, and titer were examined. To determine the effect of SCAMP3 knockdown on viral RNA synthesis, SF268 cells that had been transfected with negative-control (NC) siRNA or SCAMP3 siRNA were then infected with EV-A71/4643 at an MOI of 10. Viral RNA levels were examined by real-time reverse transcription-PCR (RT-PCR) at different time points postinfection. As shown in Fig. 3A, viral RNA levels following knockdown of SCAMP3 cells decreased approximately 68% and 49% at 8 and 10 h postinfection, respectively.

The effect of SCAMP3 knockdown on viral protein synthesis was next examined. SF268 cells were transfected with NC siRNA or siRNA against SCAMP3 and were subsequently infected with EV-A71/4643 at an MOI of 10. Cell lysates were collected at various time points and analyzed by 12% SDS-PAGE and Western blotting. The results showed that viral 3D^pol^, 3CD, and P3 levels were lower in SCAMP3 knockdown cells than in control cells by 8 h and 10 h postinfection (Fig. 3B, compare lane 7 with lane 8 and lane 9 with lane 10).

The effect of SCAMP3 knockdown on EV-A71 viral titer was also examined. SF268 cells that had been transfected with NC siRNA or SCAMP3 siRNA were then infected with EV-A71/4643 at an MOI of 10. Viral titers were examined by plaque formation assay at different time points postinfection. As shown in Fig. 3C, viral titer from knockdown of SCAMP3 cells decreased approximately 62% at 24 h. siRNA-transfected RD cells were also infected with EV-A71-3A-FLAG, and, similarly to that in SF268 cells, viral RNA, protein, and titer were lower following knockdown of SCAMP3 than in control cells (Fig. 3D to F).

To substantiate the results from siRNA-mediated knockdown, CRISPR/caspase 9 was used to generate an RD stable cell line with SCAMP3 knockout (KO). The effects of SCAMP3 knockout on EV-A71 viral RNA replication, viral protein synthesis, and viral growth were examined. Scramble or SCAMP3 KO RD cells were infected with EV-A71-3A4-FLAG at an MOI of 20, and viral RNA synthesis, viral protein levels, and virus titers were examined. As shown in Fig. 3G, viral RNA levels from SCAMP3 KO cells decreased at different time points postinfection. Viral protein levels from SCAMP3 KO cells also decreased at different time points postinfection (Fig. 3H). Viral titer from SCAMP3 KO cells decreased approximately 86% at 12 h (Fig. 3I). Taken together, these results indicate that SCAMP3 positively regulates EV-A71 replication.

### SCAMP3 associates with PI4KIIIβ, 3A and 3D^pol^ proteins in infected cells.

**FIG 4:**
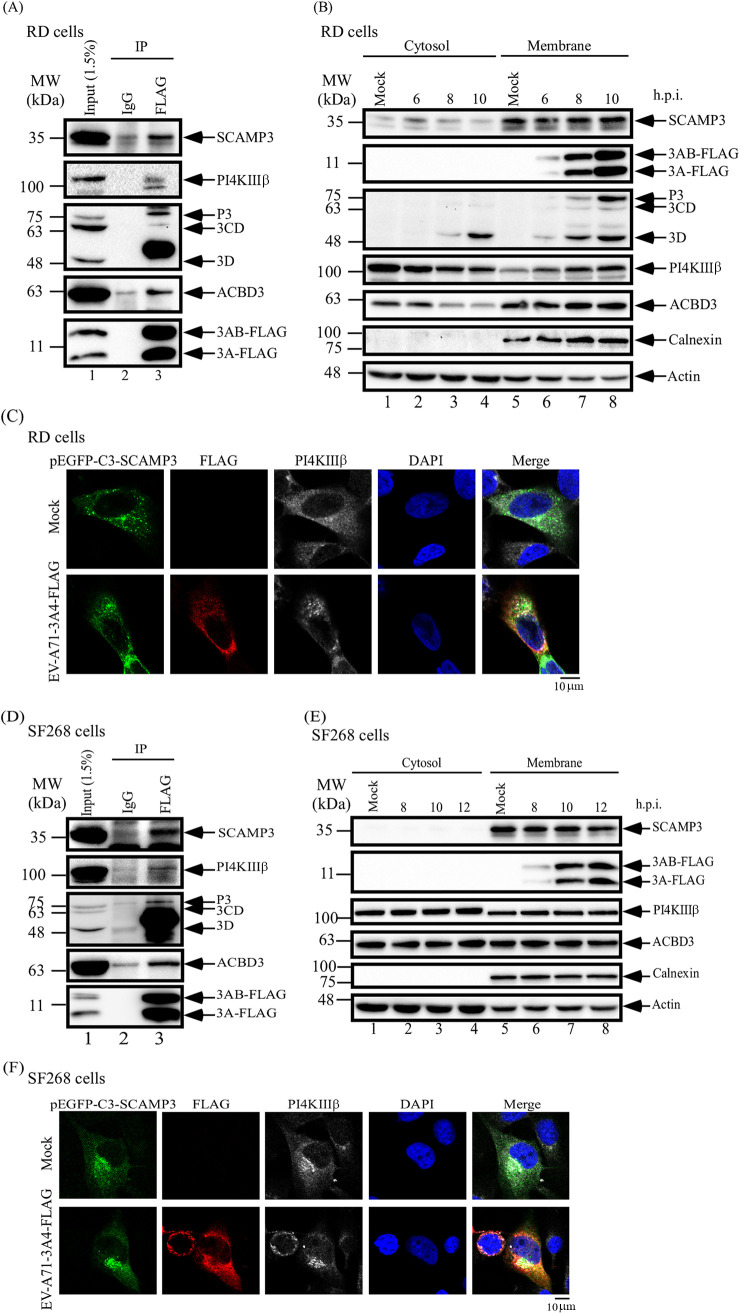
EV-A71 3A protein, SCAMP3, and PI4KIIIβ associate and colocalize in EV-A71-infected cells. (A) A co-immunoprecipitation assay was carried out. RD cells were infected with EV-A71-3A4-FLAG recombinant virus at an MOI of 20 for 6 h. Infected-cell lysates were immunoprecipitated with control IgG or anti-FLAG antibodies, and proteins that bound to the resin were analyzed by 12% SDS-PAGE, followed by immunoblotting with anti-SCAMP3, anti-FLAG, anti-3D, anti-ACBD3, and anti-PI4KIIIβ antibodies. (B) RD cells were infected with EV-A71-3A4-FLAG recombinant virus at an MOI of 20, and cell lysates were collected to isolate cytosol and membrane fractions at different time points postinfection. The fractions were analyzed for viral and host proteins levels by Western blotting. (C) SCAMP3, PI4KIIIβ, and viral 3A protein were localized to RNA replication complexes in EV-A71-infected RD cells. RD cells were transfected with pEGFP-C3-SCAMP3 for 48 h. Transfected RD cells were mock infected or infected with EV-A71-3A4-FLAG at an MOI of 20. At 6 h postinfection, cells were fixed with formaldehyde, washed, and immunostained with antibody against 3A-FLAG and PI4KIIIβ. DAPI was used to stain the nucleus. Images were captured by confocal laser scanning microscopy. (D) SF268 cells were infected with EV-A71-3A4-FLAG recombinant virus at an MOI of 20 for 12 h. Infected-cell lysates were immunoprecipitated with control IgG or anti-FLAG antibodies, and proteins that bound to the resin were analyzed by 12% SDS-PAGE, followed by immunoblotting with anti-SCAMP3, anti-FLAG, anti-3D, anti-ACBD3, and anti-PI4KIIIβ antibodies. (E) SF268 cells were infected with EV-A71-3A4-FLAG recombinant virus at an MOI of 20, and cell lysates were collected to isolate cytosol and membrane fractions at different time points postinfection. The fractions were analyzed for viral and host proteins levels by Western blotting. (F) SCAMP3, PI4KIIIβ, and viral 3A protein were localized to RNA replication complexes in EV-A71-infected SF268 cells. SF268 cells were transfected with pEGFP-C3-SCAMP3 for 48 h. Transfected RD cells were mock infected or infected with EV-A71-3A4-FLAG at an MOI of 20. At 10 h postinfection, cells were fixed with formaldehyde, washed, and immunostained with antibody against 3A-FLAG and PI4KIIIβ. DAPI was used to stain the nucleus. Images were captured by confocal laser scanning microscopy. Scale bars, 10 μm.

In cells infected with enterovirus, viral and host proteins are thought to replicate the viral genome at discrete sites. Host factors, such as ACBD3, GBF1, or Arf1, might recruit PI4KIIIβ to replication complexes to promote viral RNA synthesis. To examine whether viral 3A, viral 3D^pol^, SCAMP3, and PI4KIIIβ form a complex(es) during infection, cell lysates from EV-A71-infected RD cells were examined for co-immunoprecipitation of SCAMP3, PI4KIIIβ, and 3D^pol^ following FLAG-3A immunoprecipitation. The Western blots indicate that SCAMP3, PI4KIIIβ, and viral 3D^pol^ all form one or more complexes with viral 3A/3AB at 6 h postinfection (Fig. 4A). Similar results were obtained using lysates from EV-A71-infected SF268 cells at 12 h postinfection (Fig. 4D). In this case, ACBD3, which has been reported to associate with 3A in the replication complex, served as a positive control.

Enteroviruses induce remodeling of cellular membranes to generate a scaffold for genomic RNA replication. To further confirm that viral replication complexes reside in the membrane fraction, cytosol and membrane fractions were isolated at different time points post-EV-A71 infection and analyzed by Western blotting. The results showed that viral 3A/3AB were exclusively within the membrane fraction; viral 3D^pol^, SCAMP3, PI4KIIIβ, and ACBD3 were present in both fractions to various degrees during EV-A71 infection (Fig. 4B). However, given the localization of 3A/3AB to the membrane fraction only, the other proteins would likely have to associate with 3A/3AB via membrane-localized molecules. We obtained similar membrane/cytosol distributions of viral 3A, SCAMP3, PI4KIIIβ, and ACBD3 using lysates of EV-A71-infected SF268 cells (Fig. 4E).

Viral 3A protein plays an important role in formation of active replication sites. To further characterize the cellular localization of SCAMP3, viral 3A protein, and PI4KIIIβ in EV-A71-infected cells, their subcellular localization after EV-A71 infection was compared with that after mock infection by fluorescence microscopy. The localization of SCAMP3, 3A-FLAG, and PI4KIIIβ in RD cells at 6 h of EV-A71 infection was studied using pEGFP-C3-SCAMP3, anti-FLAG (red color), and anti-PI4KIIIβ (white color) antibodies in an immunofluorescence assay by confocal microscopy (Fig. 4). The images revealed that 3A protein, SCAMP3, and PI4KIIIβ were colocalized at 6 h postinfection (Fig. 4C). In SF268-infected cells, 3A protein, SCAMP3, and PI4KIIIβ also were colocalized at 10 h postinfection (Fig. 4F).

Taken together, these data indicate that viral 3A protein, SCAMP3, and PI4KIIIβ colocalize with RNA replication sites during EV-A71 infection in RD or SF268 cells.

### SCAMP3 affects PI4KIIIβ and PI4P recruitment and virus RNA replication.

**FIG 5:**
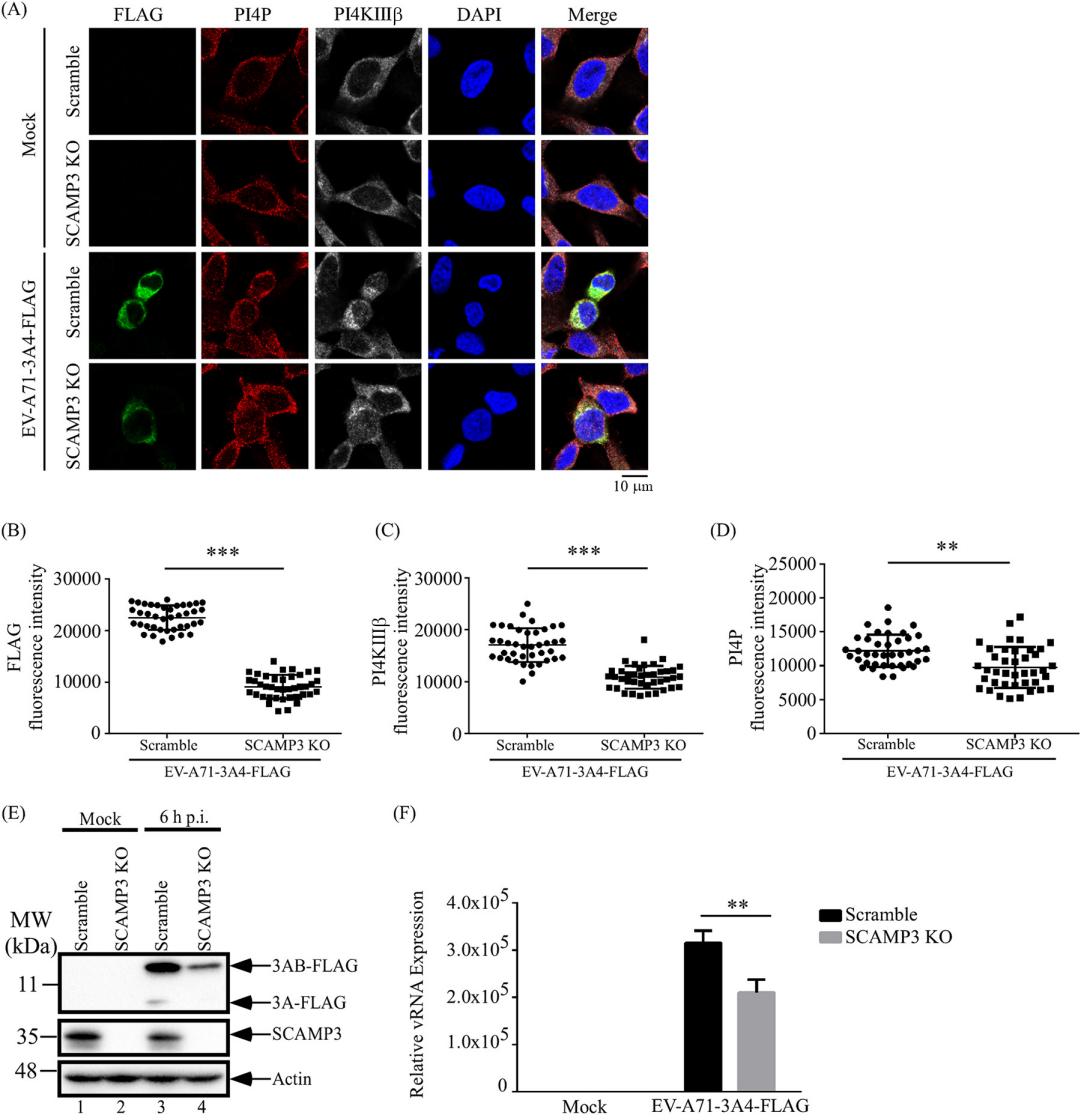
SCAMP3 affects PI4KIIIβ and PI4P recruitment for viral replication during EV-A71 infection. (A) Scramble or SCAMP3 KO RD cells were mock infected or infected with EV-A71-3A4-FLAG at an MOI of 20. At 6 h postinfection, cells were fixed with formaldehyde, washed, and immunostained with antibody against 3A-FLAG, PI4KIIIβ, and PI4P. DAPI was used to stain the nucleus. Images were captured by confocal laser scanning microscopy. (B) Quantification of the FLAG fluorescence intensity of EV-A71-infected scramble and SCAMP3 KO cells. (C) Quantification of the PI4KIIIβ fluorescence intensity of EV-A71-infected cells. PI4KIIIβ level was determined by fluorescence intensity overlap of the 3A-FLAG area of the whole cells. (D) Quantification fluorescence intensity of PI4P of EV-A71-infected cells. PI4P level was determined by fluorescence intensity overlap of the 3A-FLAG area of the whole cells. Bars represent the means from 40 cells from the experiment. Significant differences compared to EV-A71-infected scramble cells are indicated as follows: **, *P* < 0.01, ***, *P* < 0.001. (E) Western blot analysis of viral protein and SCAMP3 levels of total cell lysates. (F) Total viral RNA level was measured by RT-qPCR. Scale bar, 10 μm.

In cells infected with enteroviruses, viral 3A protein, interacting with host factors, recruits PI4KIIIβ to replication sites and may facilitate PI4P production in the replication complex and subsequent viral RNA replication. We thus examined whether 3A utilizes SCAMP3 to recruit PI4KIIIβ to the replication complex to facilitate PI4P enrichment and subsequent viral RNA replication. Scramble or SCAMP3 KO cells were infected with EV-A71 for 6 h, and cellular protein levels and localization of viral 3A-FLAG, PI4KIIIβ, and PI4P were detected by confocal microscopy. The results are shown in Fig. 5A. Forty EV-A71-infected cells were selected to quantify the fluorescence density of 3A-FLAG in order to monitor viral protein expression levels in scramble control and SCAMP3 KO cells. The results showed that fluorescence intensity of viral 3A/FLAG in SCAMP3 KO infected cells was lower than that in scramble infected cells (Fig. 5B). Total viral 3A-FLAG and 3AB-FLAG protein levels assessed by Western blotting showed comparable results (Fig. 5E). We further analyzed the fluorescence intensity of PI4KIIIβ and PI4P at 3A protein expression sites. The fluorescence intensity of PI4KIIIβ and PI4P in SCAMP3 KO-infected cells was lower than in control scramble-infected cells (Fig. 5C and D). Concomitantly, viral RNA synthesis decreased 33% in SCAMP3 KO-infected cells (Fig. 5F). These data indicate that SCAMP3 affects PI4KIIIβ and PI4P recruitment and, subsequently, viral RNA replication during EV-A71 infection.

### SCAMP3 may act through the ERK pathway, but not AKT, to promote EV-A71 replication.

**FIG 6:**
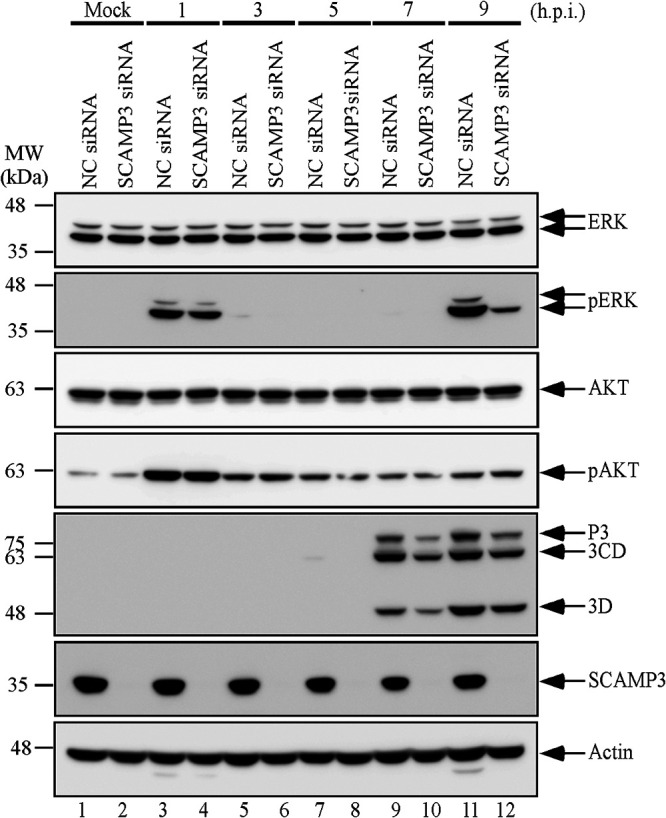
SCAMP3-depleted cells indicate the ERK pathway, but not AKT pathway, is involved in EV-A71 replication. SF268 cells were transfected with NC siRNA or siRNA against SCAMP3. Cells were mock infected or infected with EV-A71 at an MOI of 20 2 days after transfection. Total cell lysates were examined by Western blotting with anti-ERK, anti-pERK, anti-AKT, anti-pATK, anti-3D^pol^, anti-SCAMP3, and anti-actin antibodies.

EV-A71 infection activates the extracellular signal-regulated kinase (ERK) signaling pathway in a biphasic fashion; ERK activation is required for innate immune responses and for virus replication (33). Our results show that SCAMP3 positively regulates EV-A71 replication. We thus tested whether SCAMP3 serves to activate the ERK signaling pathway to promote viral replication. SF268 cells were transfected with NC siRNA or siRNA against SCAMP3 and were subsequently infected with EV-A71-3A4-FLAG at an MOI of 20. Cell lysates were collected at various time points and analyzed by Western blotting. The results showed that EV-A71 induced early activation of ERK upon SCAMP3 knockdown (Fig. 6, pERK lanes 3 and 4) but failed to fully stimulate the second phase of activation compared to that in NC siRNA-transfected cells (Fig. 6, compare pERK lanes 11 and 12). EV-A71 also induced early activation of AKT, but did not stimulate the second phase of activation (Fig. 6, compare pAKT lanes 3 and 4 and lanes 11 and 12). These data indicate that SCAMP3 may regulate the ERK signaling pathway to affect viral RNA synthesis and virus replication.

### SCAMP3 associates with 3A protein of CVB3 and enhances viral replication.

**FIG 7:**
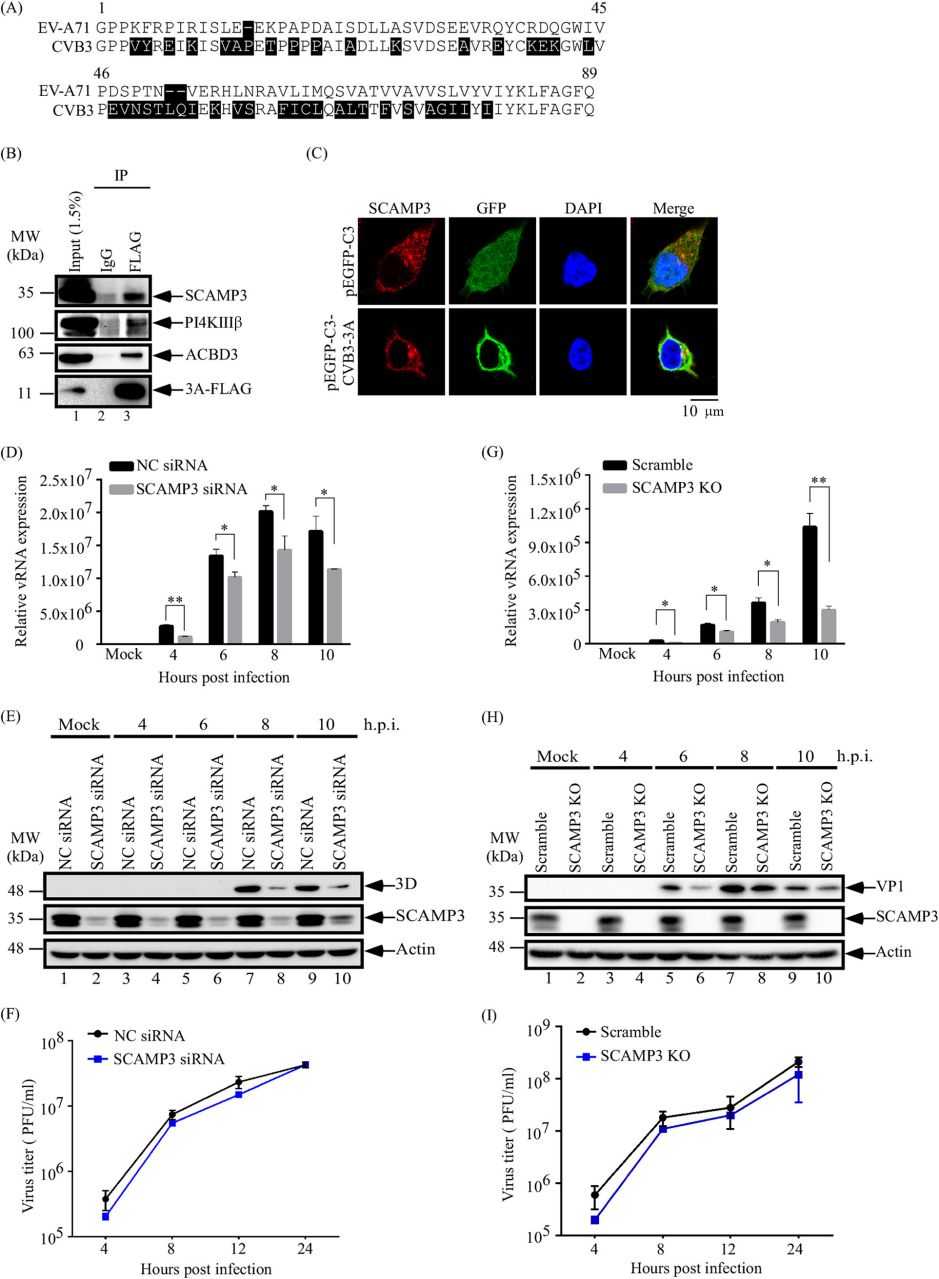
SCAMP3 associates with 3A protein of CVB3 and enhances viral RNA replication. (A) Sequence alignment of EV-A71 and CVB3 3A proteins. (B) A co-immunoprecipitation assay was carried out. RD cells were transfected with CVB3-3A-FLAG for 2 days. Transfected cell lysates were immunoprecipitated with control IgG or anti-FLAG antibodies, and proteins bound to the resin were analyzed by 12% SDS-PAGE, followed by immunoblotting with anti-SCAMP3, anti-PI4KIIIβ, anti-ACBD3, and anti-FLAG antibodies. (C) Colocalization of SCAMP3 with EGFP-C3-CVB3 3A protein. RD cells were transfected with pEGFP-C3 or pEGFP-C3-CVB3-3A. At 48 h posttransfection, cells were fixed with formaldehyde, washed, and immunostained with antibody against SCAMP3. DAPI was used to stain the nucleus. Images were captured by confocal laser scanning microscopy. (D) Effect of SCAMP3 knockdown on CVB3 RNA levels. RD cells were transfected with NC siRNA or siRNA against SCAMP3. Cells were mock infected or infected with CVB3 at an MOI of 0.1 2 days after transfection. Total RNA was extracted and viral RNA levels were determined by RT-qPCR. (E) Effect of SCAMP3 knockdown on CVB3 viral 3D^pol^ protein levels. RD cells were transfected with NC siRNA or siRNA against SCAMP3. Cells were mock infected or infected with CVB3 at an MOI of 0.1 2 days after transfection. Total cell lysates were examined by Western blotting. (F) Effect of SCAMP3 knockdown on CVB3 viral growth. RD cells were transfected with NC siRNA or siRNA against SCAMP3. Cells were mock infected or infected with CVB3 at an MOI of 0.1 2 days after transfection. Viruses were harvested at different time points postinfection and assayed by plaque formation with RD cells. (G) Effect of SCAMP3 knockout on CVB3 RNA levels. Scramble or SCAMP3 KO RD cells were mock infected or infected with CVB3 at an MOI of 0.1. Total RNA was extracted and viral RNA levels were determined by RT-qPCR. (H) Effect of SCAMP3 knockout on CVB3 viral 3D^pol^ protein levels. Scramble or SCAMP3 KO RD Cells were mock infected or infected with CVB3 at an MOI of 0.1. Total cell lysates were examined by Western blotting. (I) Effect of SCAMP3 knockout on CVB3 viral growth. Scramble or SCAMP3 KO RD cells were mock infected or infected with CVB3 at an MOI of 0.1. Viruses were harvested at different time points postinfection and assayed by plaque formation with RD cells. Scale bar, 10 μm.

To investigate whether SCAMP3 is a host factor for regulation of EV-A71 replication specifically, CVB3, a pathogen for myocarditis, was selected for study. As shown in Fig. 7A, the alignment of protein sequences between EV-A71 and CVB3 shows 48.3% identity. To confirm the interaction between the CVB3 3A protein and SCAMP3, pFLAG-CVB3-3A was transfected into RD cells. Lysates were immunoprecipitated with FLAG antibody to capture FLAG-3A, and Western blotting was performed to identify co-immunoprecipitated SCAMP3, ACBD3, and PI4KIIIβ. The results indicate that SCAMP3 associates with CVB3-3A and host proteins ACBD3 and PI4KIIIβ in one or more complexes (Fig. 7B). To characterize the cellular localization of SCAMP3 and CVB3-3A, their subcellular localization was assessed by fluorescence microscopy following transfection of pEGFP-C3-CVB3-3A or control pEGFP-C3 plasmid. The localization of EGFP-CVB3 3A and SCAMP3 in RD cells was determined using anti-SCAMP3 (red color) antibodies in an immunofluorescence assay or EGFP fluorescence for GFP-CVB3 3A; the images revealed sites of EGFP-CVB3 3A colocalization with SCAMP3 in the cytoplasm (Fig. 7C).

Effects of SCAMP3 knockdown on CVB3 viral RNA synthesis, translation, and titer were next examined. RD cells were transfected with negative-control (NC) siRNA or SCAMP3 siRNA and then infected with CVB3 at an MOI of 0.1. Samples were collected at various time points postinfection for analyses of virus RNA and protein levels and virus titers. As determined by real-time RT-PCR (Fig. 7D), viral RNA levels from knockdown of SCAMP3 cells decreased approximately 59%, 24%, 29%, and 34% at 4, 6, 8, and 10 h postinfection, respectively. Western blot analyses of lysates showed that viral protein 3D^pol^ levels in SCAMP3-depleted cells were lower than protein levels in control cells by 8 h and 10 h postinfection (Fig. 7E, compare lane 7 with lane 8 and lane 9 with lane 10). Finally, viral titers were examined by plaque formation assay at different time points postinfection. As shown in Fig. 7F, viral titers between negative-control and SCAMP3 knockdown cells were not significantly different (Fig. 7F).

We observed similar results using the SCAMP3 knockout RD cell line. Compared to CVB3-infected scramble RD cells, CVB3 viral RNA replication and viral protein synthesis decreased in CVB3-infected SCAMP3 KO cells (Fig. 7G and H). Viral titers between the scramble control and SCAMP3 KO cells were not significantly different (Fig. 7I). These data suggest that SCAMP3 associates with 3A of CVB3 and enhances viral RNA replication but has no significant effect on virus titers.

### SCAMP3 has no effect on DENV2 replication.

**FIG 8:**
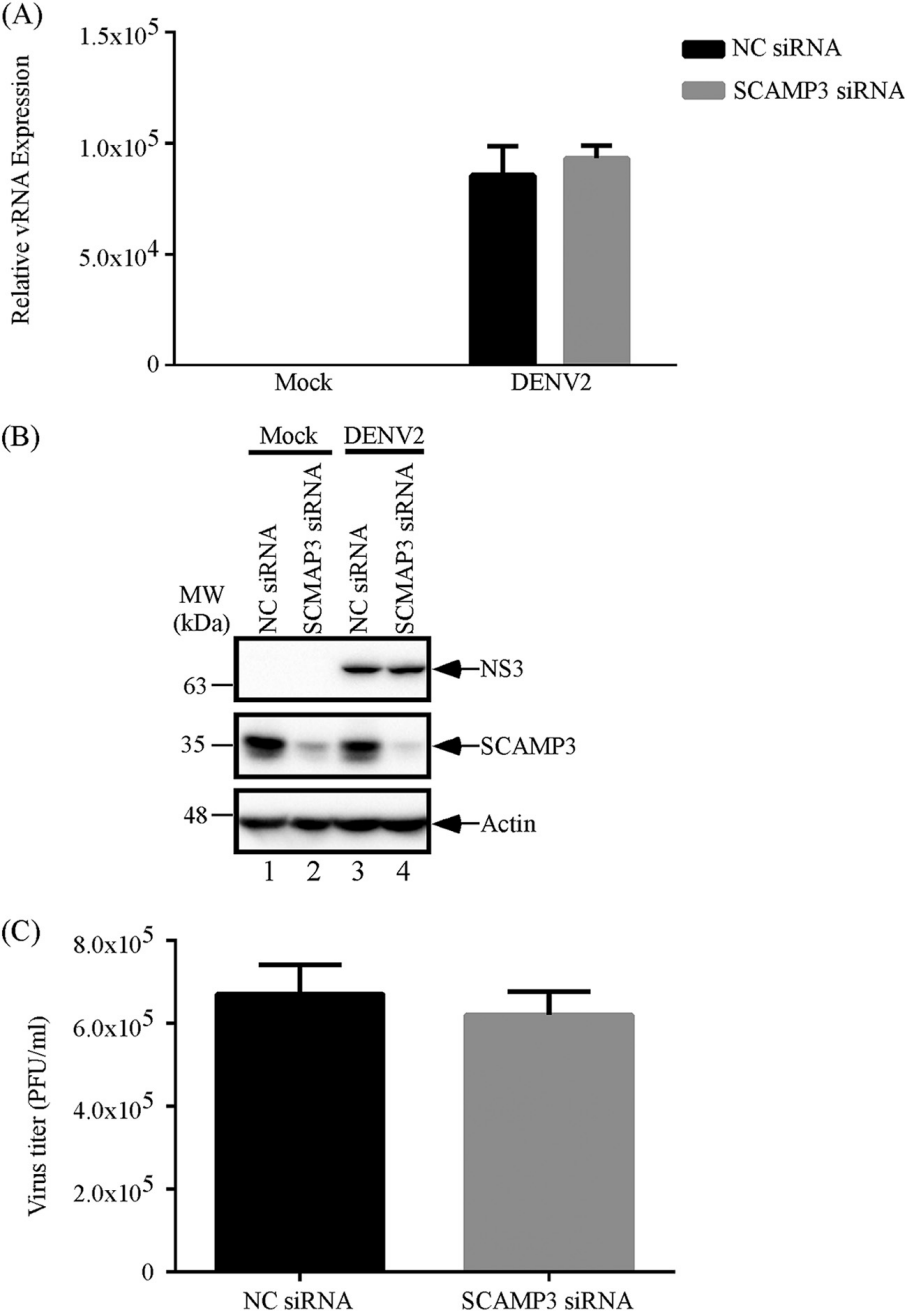
SCAMP3 does not regulate DENV2 replication. (A) Effect of SCAMP3 knockdown on DENV2 RNA levels. A549 cells were transfected with NC siRNA or siRNA against SCAMP3. Cells were mock infected or infected with DENV2 at an MOI of 1 2 days after transfection. Total RNA was extracted at 24 h postinfection, and viral RNA levels were determined by RT-qPCR. (B) Effect of SCAMP3 knockdown on DENV2 viral NS3 protein levels. A549 cells were transfected with NC siRNA or siRNA against SCAMP3. Cells were mock infected or infected with DENV2 at an MOI of 1 2 days after transfection. Total cell lysates were examined by Western blotting. (C) Effect of SCAMP3 knockdown on DENV2 viral growth. Viruses were harvested at 24 h postinfection and assayed by plaque formation with BHK-21 cells.

Not only enteroviruses interact with host factors to form replication complexes, but other RNA viruses also utilize similar mechanisms. To investigate whether SCAMP3 is an important host factor for regulating replication of other positive-strand RNA viruses, dengue virus 2 (DNEV2), a member of the *Flaviviridae* family, was selected for investigation. To examine possible effects of SCAMP3 on dengue virus 2 (DENV2) viral RNA replication, viral protein synthesis, and virus titer, A549 cells were transfected with negative-control (NC) siRNA or SCAMP3 siRNA and then infected with DENV2 at an MOI of 1; samples were collected at 24 h. As shown in Fig. 8A to C, viral RNA levels, viral protein levels, and virus titers were not significantly different between negative-control and SCAMP3 knockdown cells. Taken together with results from all three viruses we examined, we conclude that SCAMP3 affects virus replication for EV-A71 specifically.

## DISCUSSION

An important feature of enterovirus infection is the formation of new membranous structures that support viral RNA replication (13). The functionality of enterovirus replication organelles depends on the activities of both viral nonstructural proteins and co-opted host proteins. 3A proteins of enteroviruses are responsible for active recruitment of factors that support viral replication. EV-A71 was chosen here as a virus model to investigate the interactions of the EV-A71 3A protein with viral and cellular proteins in infected cells. Immunoprecipitation was combined with LC-MS/MS analysis to identify 172 cellular proteins and four viral proteins that associate with viral 3A protein (Fig. 1F and Table 1). We demonstrated by immunoprecipitation assay that SCAMP3 associates with 3A protein and it colocalizes with 3A protein during virus infection (Fig. 2). SCAMP3 knockdown or knockout in infected cells decreased synthesis of EV-A71 viral RNA, viral proteins, and viral growth (Fig. 3). SCAMP3 is a novel factor that regulates enterovirus replication. SCAMP3 is a member of the secretory carrier membrane proteins (SCAMP) family. This family consists of SCAMP1, −2 and −3, which have a long N-terminal domain with Asn-Pro-Phe (NPF) repeats, as well as SCAMP4 and −5 (34, 35). These proteins share a tetraspanning structure with cytoplasmic N- and C-terminal domains and are present in both the Golgi apparatus and endosomes (36). SCAMPs are integral membrane proteins that are present in secretory and endocytic carriers and are involved in membrane trafficking (36). The N-terminal domain of the 38-kDa SCAMP3 protein contains NPF repeats, a proline-rich segment, and a leucine repeat. The proline-rich segment contains PY, PSAP, and PTEP motifs. SCAMP3 is a putative membrane-trafficking protein that is involved in endocytosis (37). SCAMP3 also interacts with ESCRTs and is involved in epidermal growth factor receptor (EGFR) trafficking (38).

In this study, we propose two mechanisms by which SCAMP3 might regulate viral replication. In Fig. 5, SCAMP3 affected PI4KIIIβ and PI4P recruitment for EV-A71 replication. Therefore, we propose that the first mechanism is that viral 3A associates with SCAMP3 and recruits PI4KIIIβ to form replication complexes and enhances PI4P production. Then, viral 3D polymerase binds to the replication complex to generate viral RNA. Other laboratories have reported that the 3A proteins of PV, EV-A71, and CVB3 interact with host factors ACBD3 or GBF1/Arf-1 to recruit PI4KIIIβ, which is a critical host factor for viral RNA replication, to the sites of RNA replication (22, 25, 39). In enterovirus-infected cells, PI4KIIIβ is actively recruited to replication sites, generating a microenvironment that is rich in PI4P lipids. The protein c10orf76 (chromosome 10, open-reading frame 76) is a PI4KIIIβ-associated protein that increases PI4P levels at the Golgi apparatus (40, 41). This environment may attract the RNA-dependent RNA polymerase 3D^pol^ to the replication complex, because 3D^pol^ specifically binds PI4P over other cellular lipids *in vitro* (22). Finally, 3D^pol^ promotes the synthesis of viral RNA.

The MEK1-ERK signaling pathway is required for EV-A71 replication. EV-A71 induces biphasic activation of ERK1/2. The two phases of ERK1/2 activation involve different control mechanisms, with viral protein and RNA synthesis only being necessary for the second phase (33). Figure 6 shows that EV-A71 induced early activation of ERK but failed to fully stimulate the second phase of activation upon SCAMP3 knockdown in SF268 cells. Therefore, we propose that the second mechanism is that SCAMP3 is involved in the ERK phosphorylation signaling pathway to regulate viral replication. The detailed mechanism of ERK signaling requires future investigation.

All positive-strand RNA viruses induce remodeling of cellular membranes to generate a scaffold for genomic RNA replication, increase the local concentration of viral and cellular cofactors that are required for replication, and provide a protected environment that inhibits recognition of virus proteins and nucleic acid by the innate immune system. The development and functioning of these cellular membrane structures depend on reprogramming of cellular pathways into new configurations that are induced and regulated by viral proteins and host factors (13, 15). Different viruses interact with viral and host factors and use different cellular membranes to form replication complexes. In this work, we found that SCAMP3 is involved in regulating EV-A71 and CVB3 viral RNA synthesis, but SCAMP3 knockdown or knockout did not affect CVB3 virus titers. SCAMP3 has no effect on DENV2 replication (Fig. 3, 7, and 8). Thus, SCAMP3 is a novel and specific host factor for enterovirus A71 replication.

In summary, we identify SCAMP3 as a novel host factor that regulates enterovirus replication. Understanding how viral proteins hijack the regulatory mechanisms of membrane metabolism and form replication organelles will provide new insights into various areas of cell biology and virology and will be indispensable for development of a new generation of antiviral control strategies.

## MATERIALS AND METHODS

### Plasmid construction.

The EV-A71-3A-FLAG/pCR-XL-Topo plasmid encodes the full-length genome of EV-A71 with an insertion of 24 nucleotides (nt) (GACTATAAAGACGATGATGACAAG) at different position of 3A gene, as shown in Fig. 1A, with arrows indicating sites that result in the insertion of the 8-amino acid FLAG tag (DYKDDDYK) at different sites of EV-A71 protein 3A. The EV-A71-3A/GFP plasmid was constructed as follows: the 3A of EV-A71 was amplified by PCR from an EV-A71 full-length infectious cDNA clone using a 3A 5′ primer that contained a BglII site (GGAAGATCTGGGACCCCCTAAATTTAGA) and a 3A 3′ primer that contained a SalI site (CGCGTCGACTTGAAAACCGGCGAACAA). PCR products were subcloned between the BglII and SalI sites of the pEGFP-C3 vector. The SCAMP3/pAll-Cas9.Ppuro plasmid was constructed as follows: the SCAMP3 gDNA consisted of two oligonucleotides, gDNA-F (CACCGTGTGGGGCTGAGCTTTCTCG) and gDNA-R (AAACCGAGAAAGCTCAGCCCCACAC). These were 5′-end labeled using T4 polynucleotide kinase and then annealed. The annealed product was subcloned between the BsmBI sites of the pAll-Cas9.Ppuro vector.

### Cells and virus.

SF268 (human glioblastoma), RD (human embryonal rhabdomyosarcoma), BHK-21 (baby hamster kidney fibroblast), and A549 (human alveolar adenocarcinoma) cells were cultured at 37°C in Dulbecco’s modified Eagle medium (DMEM) supplemented with 10% fetal bovine serum (FBS). C6/36 cells (mosquito cell line) were cultured at 28°C in RPMI 1640 medium supplemented with 5% FBS. EV-A71 (EV-A71-3A4-FLAG recombinant virus), EV-A71/4643, and CVB3 were propagated in RD cells. DENV2 was propagated in C6/36 cells. Cells were infected with virus at the specified multiplicity of infection (MOI) and then incubated at 37°C for 1 h for adsorption. Unbound virus was removed, and cells were refed with fresh medium. Media from infected cultures were harvested at the times indicated in the text and figures, and titers of EV-A71 or CVB3 were measured by plaque formation on RD cells. The titers of DENV2 were measured by plaque formation on BHK-21 cells.

### Immunoprecipitation and LC-MS/MS.

Cell extracts from EV-A71-3A4-FLAG recombinant virus-infected RD cells, for use in immunoprecipitation assays, were prepared at 12 h postinfection (p.i.). Five milligrams of cell lysate was diluted with 450 μl lysis buffer and then added to 2 μg IgG or FLAG antibody, followed by incubation on ice for 16 h. Prewashed protein G agarose (100 μl in phosphate-buffered saline [PBS]; 50:50) was added to each sample, which was then incubated on ice for 1 h. Immune complexes were pelleted by centrifugation at 1,000 × *g* at 4°C for 5 min and washed three times with lysis buffer. After centrifugation and bead washing, the coprecipitated proteins were separated by 8% to 16% gradient SDS-PAGE, which was followed by silver staining. Proteins were identified using in-gel digestion and analyzed by LC-MS/MS. Mass lists were performed by peptide mass fingerprinting using Proteome Discoverer Daemon 1.4 software and the Swiss-Prot 2010 database.

### Viral protein expression.

RD, SF268, or A549 cells were transfected with either the negative-control (NC) siRNA (catalog number 4390843; Thermo Fisher) or SCAMP3 siRNA (AACGGAUCCACUCCUUAUAUU). After 48 h of transfection, cells were reseeded in 12-well plates for 24 h and infected with EV-A71, CVB3, or DENV2. At different time points, cells were washed twice with PBS and lysed with 100 μl CA630 lysis buffer (25 mM Tris-HCl [pH 7.6], 300 mM NaCl, 0.5% CA630, 1.5 mM MgCl_2_, 0.2 mM EDTA, 0.5 mM dithiothreitol [DTT], and 1× proteinase inhibitor) on ice for 30 min. Cell lysates were collected after centrifugation at 15,000 rpm for 10 min. Cell lysates were incubated at 95°C for 5 min in SDS loading buffer, and equal amounts of proteins were resolved by 12% SDS-PAGE. Virus proteins were detected by Western blotting.

### Western blot analysis.

Proteins were fractionated by 12% or 15% SDS-PAGE and transferred to polyvinylidene difluoride (PVDF) membranes by the wet method. PVDF membranes were blocked with Tris-buffered saline-0.1% Tween 20 (vol/vol) that contained 5% nonfat dry milk, and were probed with antibodies indicated. Primary antibodies were used at the following dilutions: anti-SCAMP3 rabbit polyclonal, 1:4,000 (GeneTex); anti-FLAG mouse monoclonal, 1:4,000 (Sigma); anti-3D mouse monoclonal, 1:4,000 (a gift from Shin-Ru Shih, Chang Gung University); anti-3A rabbit polyclonal, 1:1,000 (a gift from Jim-Tong Horng, Chang Gung University); anti-PI4KIIIβ, 1:1,000 (Merck Millipore); anti-calnexin 1:500 (Santa Cruz Biotechnology); anti-NS3, 1:5,000 (GeneTex); anti-ACBD3, 1:1,000 (Sigma-Aldrich); and anti-actin, 1:5,000 (Millipore).

### Evaluation of RNA replication by slot blotting.

RD cells were infected with EV-A71-3A4-FLAG recombinant virus at an MOI of 20 and harvested at 2, 4, 6, 8, and 10 h p.i. RNAs were extracted and dissolved in 20× SSC (1× SSC is 0.15 M NaCl plus 0.015 M sodium citrate) containing formaldehyde for 30 min at 60°C. The reaction was then loaded onto a nitrocellulose membrane in the slot-blot manifold. After washing twice, the membrane was removed, air dried, and cross-linked in a Stratalinker (Stratagene) at 200 J for 9 min. The membrane was prehybridized at 68°C for 30 min in DIG Easy Hyb (Roche). Digoxigenin (DIG)-labeled RNA probes, specific for the genome or antigenome, were produced using a DIG Northern starter kit (Roche). After addition of the probes at 100 ng/ml, the blots were incubated at 68°C for 16 h. After hybridization, the membrane was immediately submerged in a tray containing low-stringency buffer (2× SSC containing 0.1% SDS) at room temperature for 5 min with shaking. The blot was then incubated twice (15 min each with shaking) in high-stringency buffer (0.1× SSC containing 0.1% SDS) at 68°C. The membrane was then incubated with washing buffer (Roche) for 2 min at room temperature with shaking. After the membrane had been blocked with blocking solution (Roche) for 30 min, it was incubated with alkaline phosphatase-conjugated anti-DIG antibody solution for 30 min and then washed twice with maleic acid buffer (0.1 M maleic acid, 0.15 M NaCl, 0.3% Tween 20, pH 7.5; Roche). It was then equilibrated for 5 min in 20 ml detection buffer (0.1 M Tris HCl [pH 9.5], 0.1 M NaCl; Roche). Finally, chemiluminescent substrate (CDP-Star; Roche) was added, and the membrane was exposed to Kodak film.

### Quantitative real-time reverse transcription-PCR.

Mock or infected cells were harvested at various time points, and total RNA was extracted from cells using the RNeasy Plus minikit (Qiagen). Total RNA (0.5 μg) was reverse transcribed into cDNA with a high-capacity cDNA reverse transcription kit (Applied Biosystems). The cDNAs were analyzed by quantitative PCR using Fast SYBR green master mix (Applied Biosystems) and 20 pmol of each primer (for EV-A71 level: EV-A71-F, 5′-CTGTAAATCAACGATCAATAGCAG, and EV-A71-R, 5′-GTAGTTGGT CGGGTAACGAAC; for CVB3 level: CVB3-F, 5′-GGGTCACACGTCACAAGTAGTG, and CVB3-R, GTCAGCTCCAGGTCGAACC; for DENV2 level: DENV-2-F, 5′-TATCCAATGCCTCTGGGAAC, and DENV-R, 5′-TGGCTCGTAAGTGGCTTTCT; for β-actin: β-actin-F, 5′-TGGCGCTTTTGACTCAGGAT, and β-actin-R, 5′-GGGATGTTTGCTCCAACCAA). Reactions were carried out using the ABI 7500. β-Actin mRNA served as an internal control for normalization. Relative viral RNA levels were calculated by the comparative threshold cycle (2^−ΔΔ^*^CT^*) method.

### Fluorescence microscopy analysis.

RD cells grown on glass coverslips were infected with EV-A71 for 1 h at an MOI of 20. At different time points postinfection, the culture medium was removed and cells were washed and fixed. The cells were then permeabilized in 0.1% Triton X-100 at room temperature for 5 min. For SCAMP3 and EV-A71 3A-FLAG immunostaining, the samples were blocked in PBS containing 0.5% bovine serum albumin (BSA) for 60 min at room temperature and then incubated with anti-SCAMP3 antibody (1:500; GeneTex), anti-PI4KIIIβ (1:250; Millipore), anti-PI4P (1:200; Echelon Biosciences), or anti-FLAG (1:500; Sigma-Aldrich) antibody overnight at room temperature. The samples were then reacted with Alexa Fluor 594-conjugated goat anti-rabbit IgG (1:600; Thermo Fisher), Alexa Fluor 594-conjugated goat anti-mouse IgG (1:600; Thermo Fisher), Alexa Fluor 594-conjugated goat anti-mouse IgM (1:600; Jackson ImmunoResearch), Alexa Fluor 488-conjugated goat anti-rabbit IgG (1:600; Thermo Fisher), or Alexa Fluor 647-conjugated goat anti-mouse IgG (1:600; Thermo Fisher) for 1 h at room temperature. After washing with PBS, the samples were treated with the nuclear stain 4′,6-diamidino-2-phenylindole (DAPI) (1:500; Thermo Fisher) for 15 min and washed again three times with PBS. Images were captured using a confocal laser-scanning microscope, Zeiss LSM 880. Quantitative analyses of images were perfotmed by Zeiss software Zen 2.5.

### Cytosol and membrane protein fractionation.

Cytosol and membrane proteins were harvested with the Mem-PER Plus membrane protein extraction kit from thermo Scientific. Cells were scraped from the 6-well plate and transferred to a 15-ml tube. Cells were centrifuged at 300 × *g* for 5 min at 4°C. The cell pellet was washed with 3 ml cell wash solution and centrifuged at 300 × *g* for 5 min at 4°C. The supernatant was removed, and the cell pellet was resuspended in 1.5 ml of cell wash solution and transferred to a 2-ml microcentrifuge tube. This was centrifuged at 300 × *g* for 5 min, and the supernatant was discarded. Permeabilization buffer (180 μl) was added to the cell pellet, which was vortexed briefly to obtain a homogeneous cell suspension. This was incubated 10 min at 4°C with constant mixing. Permeabilized cells were centrifuged at 16,000 × *g* for 15 min at 4°C. The supernatant containing cytosolic proteins was carefully removed and transferred to a new tube. One hundred twenty microliters of solubilization buffer was added to the pellet and resuspended by pipetting. Tubes were incubated 30 min at 4°C with constant mixing and then centrifuged at 16,000 × *g* for 15 min at 4°C. The supernatant containing solubilized membrane and membrane-associated proteins was transferred to a new tube. The proteins from cytosolic and membrane fractions were stored at −80°C.

### Statistical analysis.

All data are presented as the means ± standard deviations (SDs) from three independent experiments, and the two-tailed Student's *t* test was used to determine statistically significant differences.
